# Discovery of Small-Molecule Inhibitors of the KCNQ1/Kv7.1 Potassium Channel with Virtual Screening and Functional Validation in Electrophysiological Assays and KCNQ1-Knockout Neural Stem Cells

**DOI:** 10.1021/acschemneuro.6c00197

**Published:** 2026-05-21

**Authors:** Kazi Asraful Alam, Josep Martí-Solans, Dorothea Schall, Sumit Kumar, Shahid Muhammad Iqbal, Knut Teigen, Simone Berkel, Daniela Mauceri, Bengt Erik Haug, Sara I. Liin, Timothy Lynagh, Aurora Martinez, Jan Haavik

**Affiliations:** † Department of Biomedicine, 60518 University of Bergen, Bergen 5007, Norway; ‡ 1658 Michael Sars Centre, University of Bergen, Bergen 5006, Norway; § Institute of Human Genetics, 235723 Heidelberg University, Heidelberg 69120, Germany; ∥ Department of Chemistry, 1658 University of Bergen, Bergen 5007, Norway; ⊥ Department of Neurobiology, Interdisciplinary Centre for Neurosciences (IZN), 7891 Heidelberg University, Heidelberg 69120, Germany; # Institute of Anatomy and Cell Biology, Dept. Molecular and Cellular Neuroscience, University of Marburg, Marburg 35037, Germany; ∇ Department of Biomedical and Clinical Sciences, Linköping University, Linköping 58185, Sweden; ○ Neuro-SysMed, Department of Neurology, Haukeland University Hospital, Bergen 5009, Norway; ◆ Division of Psychiatry, Haukeland University Hospital, Bergen 5009, Norway

**Keywords:** KCNQ1/KCNE1, virtual screening, drug discovery, ion channels, benzodiazepines, arrhythmia, neurite growth

## Abstract

Voltage-gated potassium channel KCNQ1 (Kv7.1) plays a critical role in electrical excitability in the heart, gut, and brain. Together with the auxiliary subunit KCNE1, KCNQ1 generates a slow delayed rectifier current (*I*_*Ks*_) that is essential for cardiac repolarization. Mutations and dysregulation of this channel are found in channelopathies leading to sudden death, long-QT syndrome, atrial fibrillation, epilepsy, deafness, diabetes, and neuropsychiatric disorders. Although KCNQ1 and related potassium channels are promising therapeutic targets, there are few potent, selective, and therapeutically safe inhibitors and activators available for these proteins. A virtual screening of 36,374 compounds was conducted against KCNQ1, followed by *in silico* analyses that identified eight potential ligand candidates for experimental evaluation using human KCNQ1 coexpressed with KCNE1 in *Xenopus laevis* oocytes. Electrophysiological recordings showed that the benzodiazepine-based ligand Zinc13732787 was a potent inhibitor of the channel complex, without affecting KCNQ2/KCNQ3. Based on virtual screening and molecular docking, the 1-(3-chlorophenyl)-urea substituent on the benzodiazepine core is important for selective inhibition of KCNQ1/KCNE1, as further supported by structure-activity relationship and stereochemical exploration of Zinc13732787. Furthermore, low concentrations of Zinc13732787 reduced neurite outgrowth in human neuronal stem cells (NSCs), mirroring the phenotype observed in homozygous *KCNQ1*-knockout cells. Importantly, Zinc13732787 did not affect NSC proliferation, nor did it induce cytotoxicity. In homozygous *KCNQ1*-knockout NSCs, compound Zinc13732787 had no effect on neurite outgrowth, indicating high target specificity. These findings suggest that this compound is a valuable tool for investigating the physiological and pathological roles of KCNQ1 across various tissues. Additionally, it could be used as a precursor for novel antiarrhythmic agents as well as for epilepsy and neuropsychiatric conditions.

## Graphical abstract

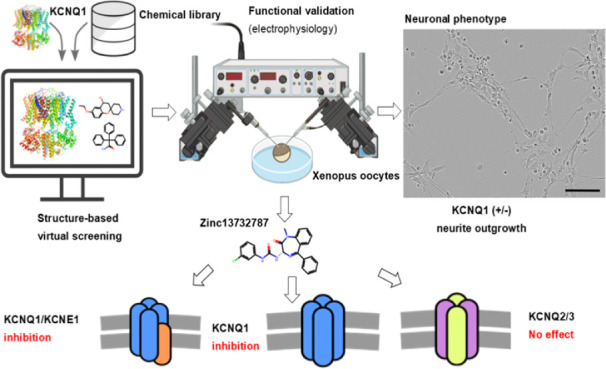

## Introduction

KCNQ1 (Kv7.1) belongs to the KCNQ family (Kv7.1–7.5) of voltage-gated potassium ion channels serving a crucial function in the electrical signaling of excitable cells.^1–3^ High expression of KCNQ1 is observed in the heart, kidney, pancreas, and auditory tissues,^4–10^ with lower levels in the brain.^11^ Other members of the KCNQ family, such as KCNQ2-4, are widely expressed in the central nervous system (CNS). Mutations in these channels are associated with neurological diseases, including epilepsy.^11^ KCNQ1 encodes a pore-forming α subunit that forms complexes in association with single-transmembrane β subunits (KCNE1) resulting in the formation of *I*_*Ks*_ current, which is important for the repolarization of the cardiac action potential.^5,6^ KCNQ1 channel activity is modified through interactions with various regulatory molecules, including phosphatidylinositol 4,5-bisphosphate (PIP2) and calmodulin.^4,12–15^ KCNQ1 represents the canonical structure of a domain-swapped, homotetrameric voltage-gated (Kv) channel, featuring four identical subunits consisting of six transmembrane domains (S1–S6), which associate with KCNEs (KCNE1–5) during assembly.^4,15,16^ The α subunit consists of the S1–S4 segments, which form the voltage-sensing domain (VSD), and S5–S6, which form the pore domain (PD).^15^ The PD controls the flow of K^+^ ions and contains the signature sequence of “Thr-Ile-Gly-Tyr-Gly” for K^+^ selectivity.^17^ KCNQ1 is associated with any of five KCNE subunits, enabling it to perform a variety of physiological functions. Binding KCNE1 increases the conductance of the channel, slows both activation and deactivation, and eliminates inactivation. Conversely, when KCNQ1 is paired with KCNE2 and KCNE3, it generates a constitutive current, while KCNE4 and KCNE5 exert inhibitory effects on KCNQ1.^12,18^ Pathogenic variants of KCNQ1 or pharmacological interference with the channel can lead to various human diseases. KCNQ1 variants are implicated in deafness and severe cardiac conditions such as long QT syndrome (LQTS),^19^ short-QT syndrome (SQTS),^20^ and atrial fibrillation (AF).^11^ Additionally, KCNQ1 overexpression inhibits insulin secretion,^21^ and its dysregulation is associated with type 2 diabetes (T2DM), gestational diabetes (GDM), epilepsy,^22^ obsessive-compulsive disorder (OCD), and Alzheimer’s disease (AD).^11,23–25^ This spectrum of cardiac and neurological involvement exemplifies heart–brain comorbidities associated with KCNQ1 variants, underscoring the complexity and systemic nature of its pathophysiology.

The cryo-EM structure of the human KCNQ1 complexed with KCNE3 and CaM (PDB ID 6V00) shows that in the absence of PIP2, KCNE3 stabilizes the voltage sensor of KCNQ1 in a depolarized conformation, while the pore remains closed.^4^ This conformation is referred to as the “activated-closed” state (PDB ID 6V00).^4^ Upon binding of the PIP2, the channel opens the pore, representing “activated-open” state, as observed in another cryo-EM structure (PDB ID 6V01).^4^ Due to the role of KCNQ1 in various human diseases, it is considered a promising therapeutic target for small molecule intervention. However, so far, few such drugs have been available, and their use has been limited due to their low selectivity and the risk of serious side effects. Access to new structural data and improved computational tools provide new opportunities to discover novel and more selective ligands targeting different members of this family of potassium channels. To this end, we performed a virtual screening of 36,374 compounds to discover ligands for KCNQ1 and tested the activity of eight potential binders in KCNQ1 channels expressed in *Xenopus laevis* oocytes. We found that Zinc13732787, i.e. (1-(3-chlorophenyl)-3-[(3*S*)-1-methyl-2-oxo-5-phenyl-3*H*-1,4-benzodiazepin-3-yl]­urea), modulates KCNQ1/KCNE1 function by decreasing its current amplitude, while having no effect on KCNQ2/KCNQ3 channels. In addition to its effects on the cardiac KCNQ1/KCNE1 complex, Zinc13732787 inhibited neurite outgrowth of human iPSC-derived neuronal stem cells at low concentrations consistent with previous findings of pharmacological inhibition by JNJ303 and in KCNQ1 knockouts.^26^

## Results

### Binding Site Analysis

The KCNQ1 structures (PDB IDs 6V00, 6V01, and 6UZZ) were first analyzed to identify potential druggable sites on KCNQ1 that could accommodate small molecule ligands, we first carried out a binding site analysis using the P2Rank,^27^ FTSite,^28^ and Site-map^29^ prediction tools. These predicted binding pockets (Figure 1) provided the basis for molecular docking.

**Figure 1:**
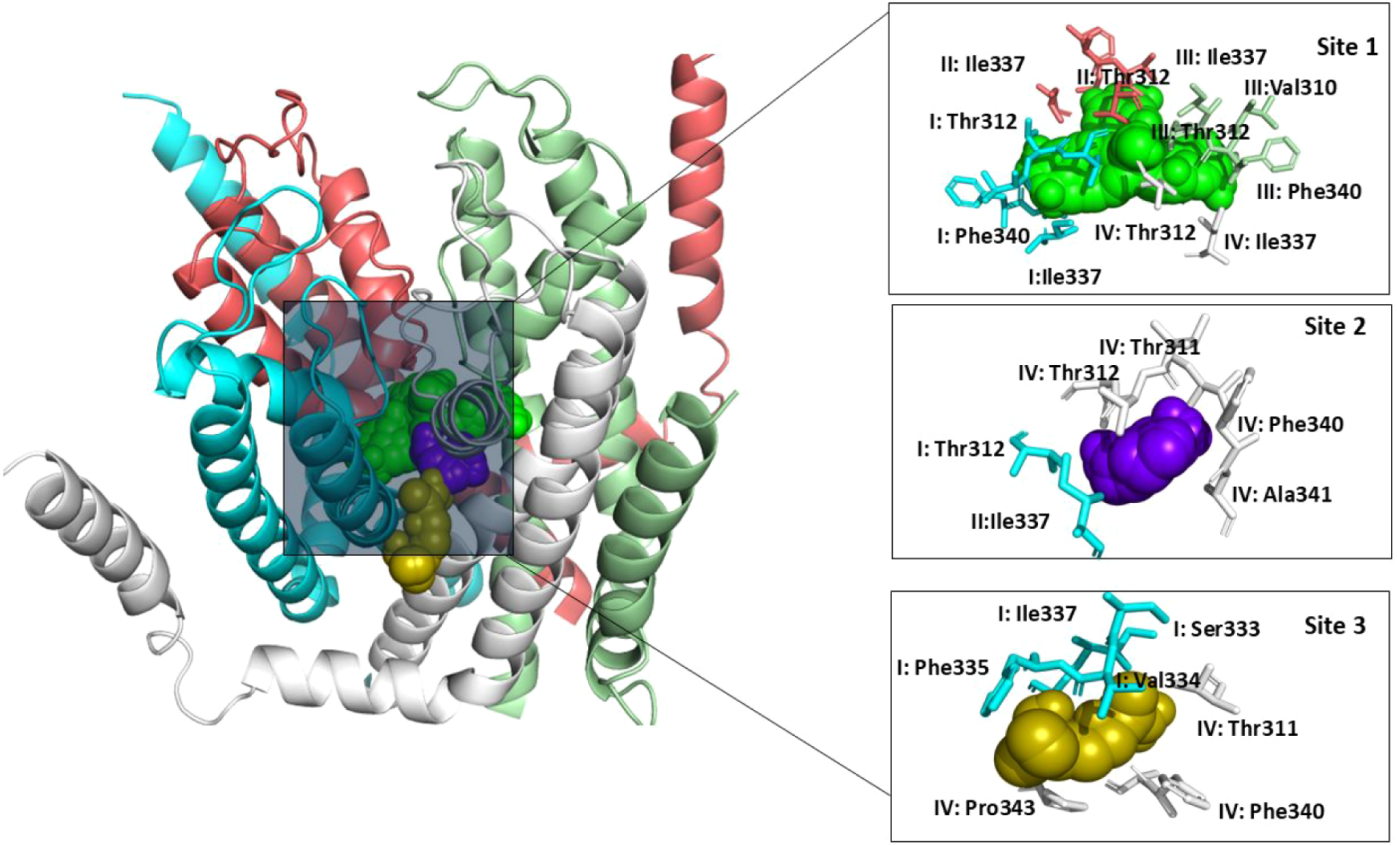
Predictive ligand binding sites of KCNQ1. KCNQ1 subunits (PDB ID 6V01) are shown as a cartoon presentation, with subunit I in cyan, subunit II in deep salmon, subunit III in pale green, and subunit IV in gray (left panel). Three predicted binding hotspots of KCNQ1 (Sites 1–3) are illustrated (right panel), with Site 1 in green, Site 2 in purple, and Site 3 in olive spheres. Interacting residues are shown in stick representation. Binding Site 1, positioned just beneath the pore, includes key residues Thr312, Ile337, and Phe340 from subunits I and II, as well as Thr311 and Phe340 from subunit III. Binding site 2 displays interactions from Thr312, Phe340, and Ala341 from subunit IV, along with Thr312 and Ile337 from subunit I. Binding site 3 features predicted interactions with Ser333, Val334, and Phe335 from subunit I, and Thr311 and Thr340 from subunit IV. Only key residues are shown here for clarity.

Predicted binding site 1 is located at the center of the pore and is defined by amino acid residues from the respective subunits of KCNQ1.^28^ These residues include Val310, Thr311–312, Ile337, Phe340, and Ala341 from subunits I, II, and III, as well as Thr312 and Ile337 from subunit IV. Site 2 is defined by Thr312 and Ile337 from subunit I and Val310, Thr311–312, Ile337, Phe340, and Ala341 from subunit IV. Site 3 is defined by Ser333, Val334, Phe335, Ile337, and Ser338 from subunit I and Thr311, Phe340, Ala341, and Pro343 from subunit IV. Previous studies on KCNQ1 indicate that KCNQ1 inhibitors interact in the channel pore with residues Thr311–312, Ile337, and Phe340 through hydrophobic interactions.^30,31^ Chromanol 293B is a KCNQ1 inhibitor that primarily interacts with Thr312, Phe340, and Ile337 from four subunits, as supported by mutagenesis studies of KCNQ1.^30,32^

### Molecular Docking and Hit Selection

Building on the identified binding pockets, we next aimed to screen a diverse chemical space to identify small molecule modulators of KCNQ1. The 17 Å receptor grid covered all three predicted binding pockets. We carried out structure-based virtual screening against KCNQ1-apo using four chemical libraries; Prestwick chemical library (PCL, https://www.prestwickchemical.com/; Neuronal signaling library (NSL), ZINC20 *in vivo* subset, and KCNQ1-targeted library) (Figure 2).^33^ In total, 36,374 compounds were first subjected to high-throughput virtual screening (HTVS) followed by standard precision (SP) docking and Prime-MM-GBSA, resulting in 136 putative ligands to be analyzed. During manual inspection, favorable interactions within the pore and residues Thr311, Thr312, Ile337, or Phe340 were set as a criterion. After evaluating docking poses manually, considering free energy, new chemotypes, and absorption, distribution, metabolism, excretion, and toxicity (ADMET) analysis, eight ligands (Figure 2) were selected for experimental testing in *Xenopus laevis* oocytes. These ligands were prioritized based on relative free energy Δ*G* (bind), and ADMET properties (Supporting Information, Table S1).

**Figure 2:**
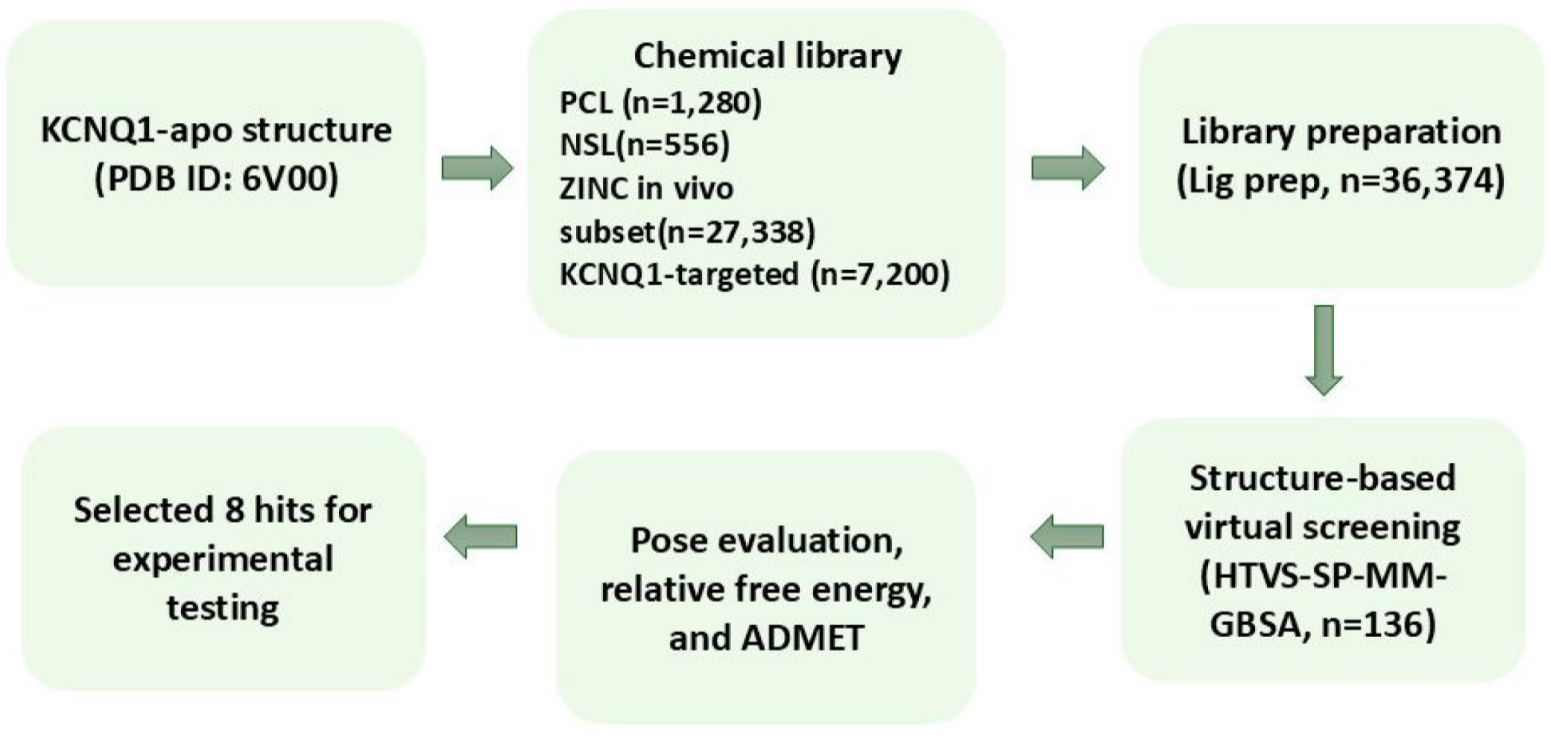
Overview of the virtual screening workflow (VSW) for KCNQ1 molecular docking. Compounds were prepared for docking into the KCNQ1 binding pocket. Docked poses were selected based on docking score, poses, Δ*G* (bind), and ADMET properties.

### Analysis of Pharmacokinetics (PK) Properties

We analyzed the predicted pharmacological and pharmacokinetic (PK) properties^34,35^ of the selected ligands with respect to cytochrome P450 (CYP1A2) and human Ether-à-go-go-Related Gene (hERG) inhibition, and their role as P-glycoprotein (P-gp) substrates using previously published prediction tools (Figure 3A, Supporting Information, Table S2). Except for Zinc95929294, all other compounds were predicted to cross the blood–brain barrier (BBB). Additionally, our analysis based on the SwissADME server^34^ revealed that out of the eight ligands, only Zinc5849645 and Zinc36141291 are predicted to interact as P-gp substrates, while the remaining compounds showed no predicted interaction. Furthermore, Zinc33819213 was predicted to inhibit cytochrome P450 enzymes.^34^

**Figure 3:**
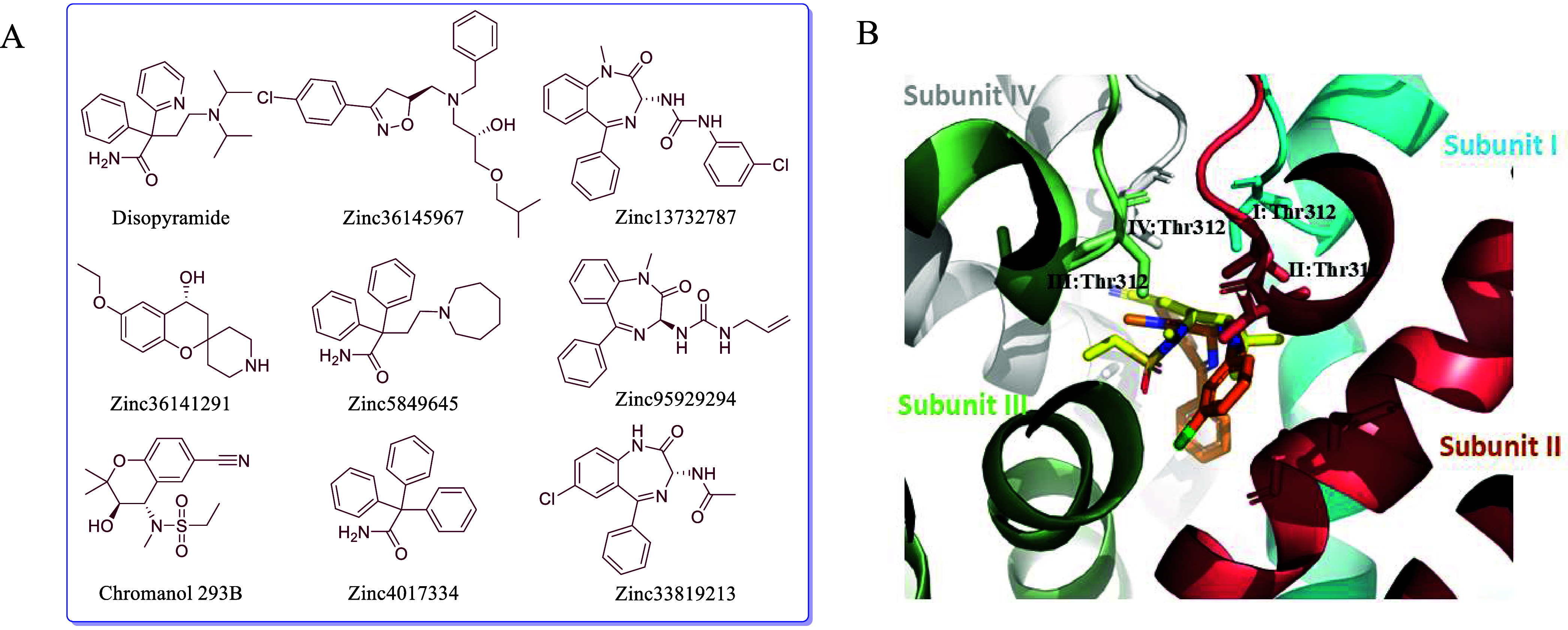
Chemical structures of eight ligand hits and overlay of the ligand binding poses from the virtual screening targeting KCNQ1-apo. A, Structures of eight selected ligands from virtual screening, including Chromanol 293B. B, Binding poses of the ligands Zinc13732787 (orange sticks) and Chromanol 293B (yellow sticks). The selectivity filter, positioned just above the ligands, is highlighted in cyan, deep salmon, pale green, and white gray, corresponding to subunits I, II, III, and IV, respectively.

### KCNQ1-Ligand Interaction

We examined docking poses of the top-scoring ligands and selected hits from the virtual screening against the KCNQ1. The poses revealed that the ligands are situated in the cavity (Figure 3B) just below the selectivity filter. The ligands are positioned around Thr311, Thr312, Ile337, Phe340, and Ala341, showing a similar binding mode to chromanol 293B in simulation and mutational studies.^30,32^

The predicted pose of Zinc36145967 has a favorable H-bond interaction with Thr312 in subunit III. Zinc36145967 is a benzyl chlorophenyl-based ligand, and the chlorophenyl moiety is oriented toward the hydrophobic patch, defined by the Ala341, Ala344, and Ile337 residues (Figure S1).

Zinc13732787 is a benzodiazepine-based ligand, where the two −NH moieties of the urea group were predicted to interact with Thr312 in subunits I and II through a hydrogen bond network (Figure S2). The predicted relative binding free energy from MM-GBSA was −26.18 kcal/mol. The benzene group and chlorobenzene revealed hydrophobic interactions with their surroundings, especially with Ile337, Phe340, and Ala341 from subunit I. The benzodiazepine moiety was predicted to be trapped in a hydrophobic patch composed of Ile337, Ala341, Ala344, and Gly345 from subunits I and IV.

Disopyramide, classified as a monocarboxylic acid amide, serves as an antiarrhythmic drug that specifically targets sodium channels.^36^ In the binding poses with KCNQ1, disopyramide is predicted to form hydrogen bond interactions with Thr312 from subunits I, II, and IV (Figure S3). A similar hydrogen-bonding network is observed for the ligand Zinc5849645 (Figure S4). In contrast, Thr311 and Thr312 from subunit III appear to interact with the ligand Zinc95129294 (Figure S5), and Zinc36141291 interacts with Thr312 from subunits II and IV. Additionally, Ser338 from subunit I interacts with this ligand (Figure S6). Zinc4017334 makes a hydrogen bond with Thr312 from subunits III and IV (Figure S7). In the docking pose of Zinc33819213, the chlorobenzene is oriented toward Ala310 of subunit I (Figure S8), which may indicate a possible interaction.

### Chemistry

The hit compound Zinc13732787 along with the *S*-enantiomer ((*S*)**−2**) and the racemate **2** were prepared from the *R*- and *S*-enantiomers or the racemate of intermediate **1**, respectively by reaction with 1-chloro-3-isocyanatobenzene in anhydrous THF (Scheme 1). Further, to examine and compare the impact of an amide and urea linker between the benzodiazepine core and the chlorobenzene substituent, **1** was treated with 2-(3-chlorophenyl) acetic acid using EDC/HOBt as coupling reagents to facilitate the formation of **3** in high yield.

**Scheme 1:**
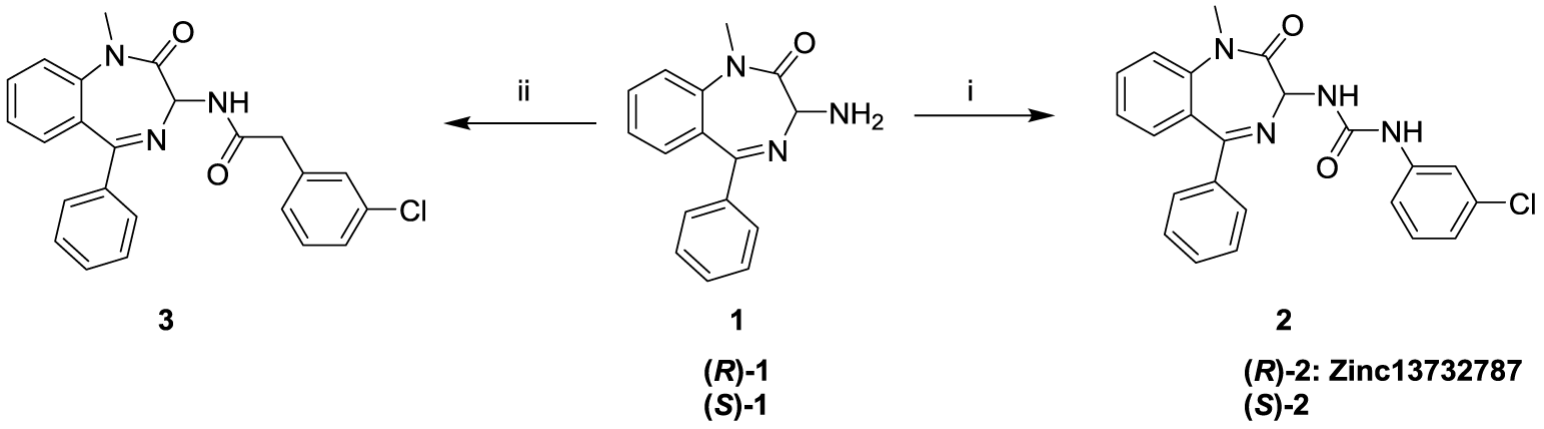
Synthesis of KCNQ1/KCNE1 Inhibitors^a^

a Reagents and conditions: (i) 1-Chloro-3-isocyanatobenzene, anhy. THF, rt, 16 h, 60–70%; (ii) 2-(3-Chlorophenyl acetic acid, EDC, HOBt, Et_3_N, DMF, rt, 12 h, 73–88%.

### Hit Validation by Two-Electrode Voltage Clamp

We next assessed whether the application of these eight ligands alters the properties of the KCNQ1/KCNE1 channel expressed in *Xenopus laevis* oocytes using the two-electrode voltage clamp (TEVC) technique. All ligands were applied at a 20 μM final concentration, and three parameters of the channel were measured: current at a depolarizing voltage of +40 mV in the presence of the drug compared to without the drug, “remaining current”; half-maximal voltage, V_50_, in the presence of the drug; and activation time constants, τ_+40_, in the presence of the drug. We used Chromanol 293B as a reference inhibitor of KCNQ1/KCNE1 channels.^32,37^

Of the tested compounds Zinc13732787 showed the strongest effect, reducing the current amplitude of the KCNQ1/KCNE1 channels by half (remaining current = 47.9 ± 9.6%; *n* = 3) compared to control (Table 1, Figure 4A). This effect is similar to the application of 20 μM Chromanol 293B, which reduced the current amplitude to 44.7 ± 3.7% (*n* = 3) (Figure 4A).

**Table 1:**
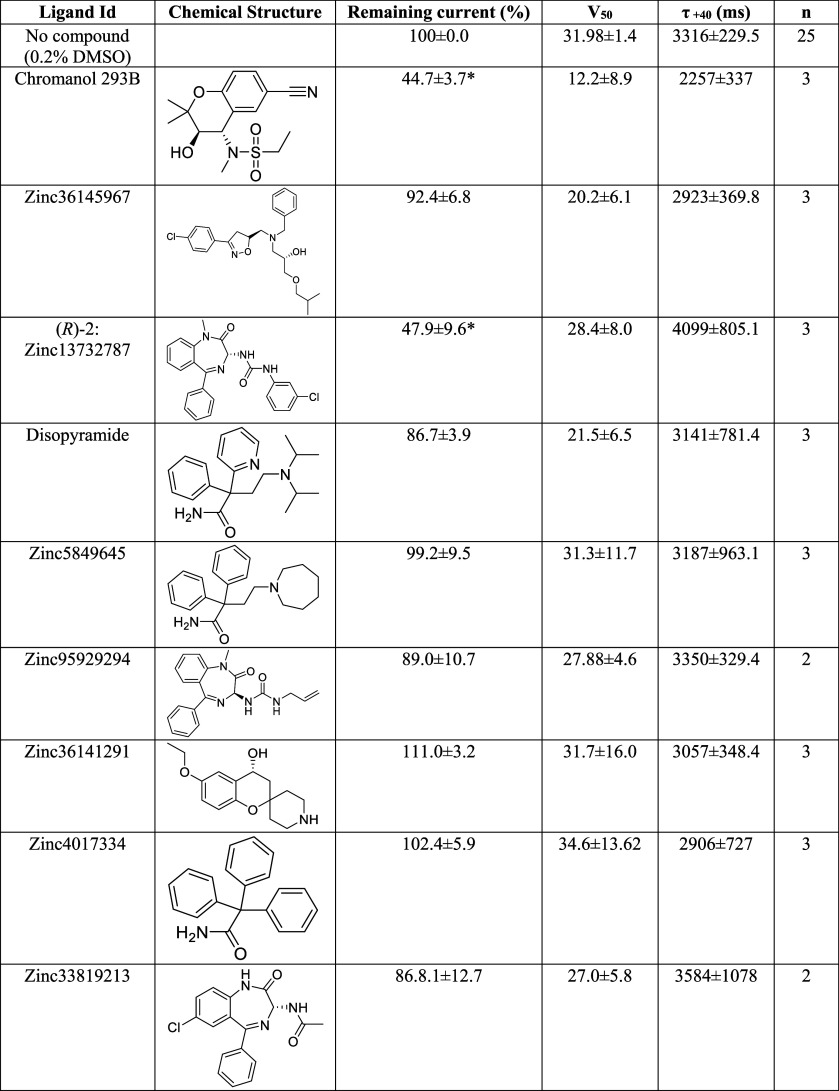
Parameters Measured for KCNQ1/KCNE1 Channel after the Application of 20 μΜ of the Selected Ligands^a^

a Current remaining after drug application (%); half-maximal voltage, V_50_; and activation time constants, τ + 40. Data were analyzed using a one-way ANOVA followed by Dunnett’s multiple comparisons test, comparing each drug treatment to the control. For remaining current *F*_(8,34)_ = 37.4, *p*-value < 0.0001 [*]; V_50_ *F*_(8,41)_ = 0.88; τ + 40, *F*_(8,40)_ = 0.71. The number of tested oocytes is denoted by *n*.

**Figure 4:**
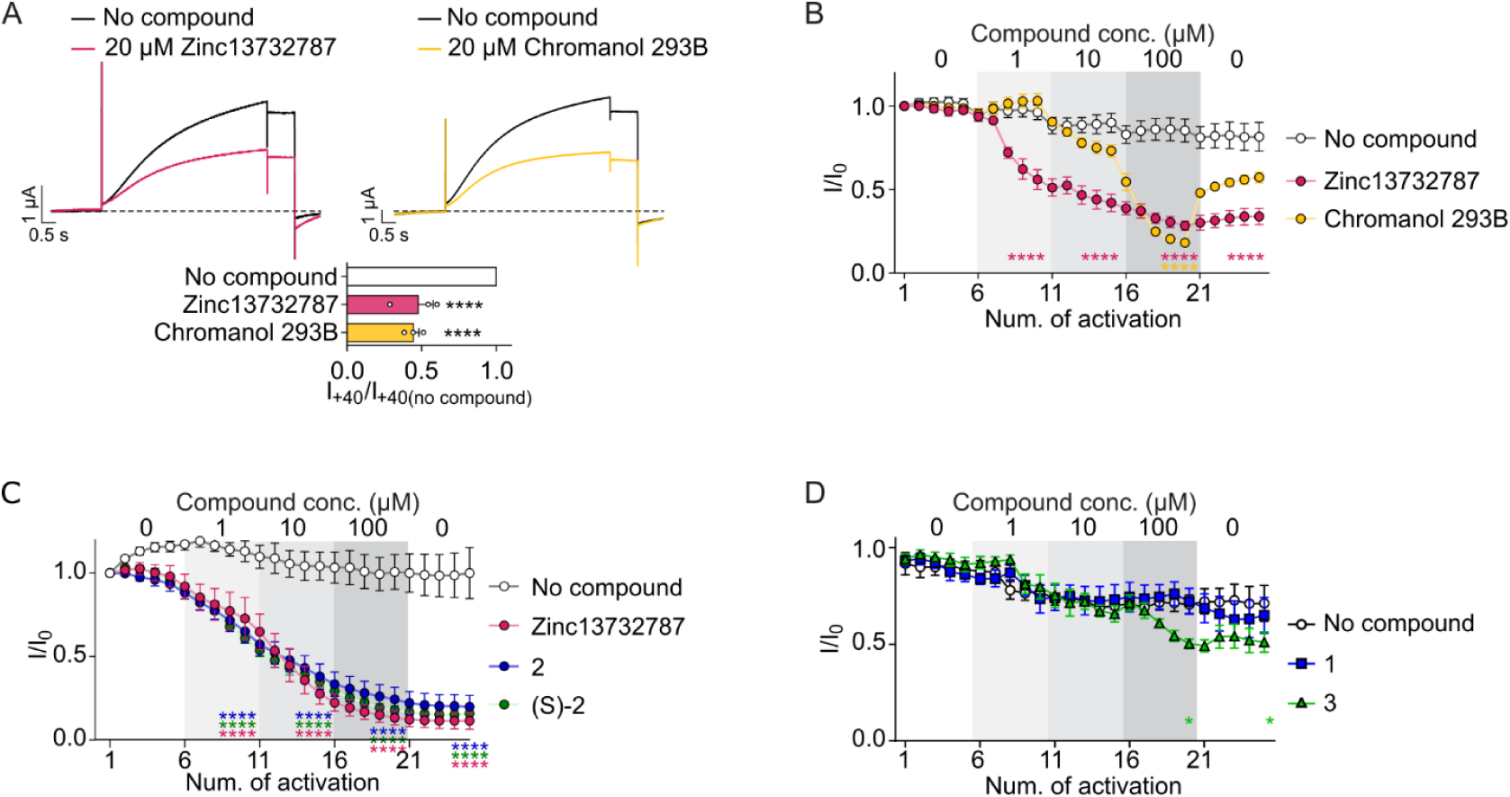
Zinc13732787 inhibits KCNQ1/KCNE1 channels. A, Example recordings (top) and averaged, normalized (bar graph) K^+^ currents through KCNQ1/KCNE1 channels activated by +40 mV in the absence or presence of indicated drugs (20 μM). B–D, Averaged, normalized K^+^ currents through KCNQ1/KCNE1 channels in response to +40 mV in the absence of drugs or in increasing concentrations of indicated drugs (resting 10 s between activations). One-way ANOVA Dunnett’s multiple comparisons test, *p < 0.05, ****p < 0.0001 compared to “no compound” after five activations, *n* = 3 (no compound in C, Chromanol 293B in B), 4 (Zinc13732787 in B and C, 3 in D, no compound in D), 5 (1 in D), 6 (no compound in B, (*S*)-2 in C), or 8 (Zinc13732787 in C). Zinc13732787 data in (B) and (C) are from different batches; data in (C) were obtained on the same day as additional compounds.

The ligands Disopyramide, Zinc36145967, Zinc95929294, and Zinc33819213 all exhibited moderate inhibition (ranging from 7.6% to 16.6%); however, this inhibition was not statistically significant, indicating a limited impact on channel function. Zinc5849645 showed minimal inhibition, with changes below 10% across the three analyzed parameters, and this lack of inhibition was also not statistically significant, indicating a negligible impact on the channel. Zinc36141291 and Zinc4017334 showed slight, but statistically nonsignificant, enhancement of the channel’s current.

Since Zinc13732787 emerged as a significant inhibitor, we measured its inhibitory potency relative to Chromanol 293B. We observed some rundown currents through KCNQ1/KCNE1 over 20 activations (white symbols in Figure 4B) making a thorough concentration–response experiment difficult. We therefore determined potency by measuring the remaining current after five activations using three different drug concentrations (Figure 4B). This showed that although 100 μM Chromanol 293B was a stronger inhibitor than 100 μM Zinc13732787, the latter showed more and near-maximal inhibition already at 10 μM (Figure 4B), suggesting this compound is a more potent KCNQ1/KCNE1 inhibitor than the reference compound Chromanol 293B. Moreover, the inhibitory effect of Zinc13732787 persisted after washout, in contrast to Chromanol 293B, whose inhibition significantly decreased after washout (Figure 4B).

In addition to testing Zinc13732787 which is the *R*-enantiomer, we synthesized the *S*-isomer **(** ***S*** **)-2** and racemic **2** and tested these against KCNQ1/KCNE1 channels. We found that currents were inhibited to 73 ± 13%, 65 ± 7%, and 61 ± 3% of their initial amplitude after five activations in the presence of 1 μM **2**, Zinc13732787, and **(** ***S*** **)-2**, respectively (*n* = 4, 8, and 6, all significantly different from the no-compound control (*p* < 0.0001), all not significantly different from each other, Figure 4C). Similarly, currents were reduced to 13 ± 4%, 24 ± 7%, and 18 ± 3% of their initial amplitude by 100 μM **2**, Zinc13732787, and **(** ***S*** **)-2**, respectively, and in each case, five subsequent activations without the tested compound showed no visible washout of the inhibition (Figure 4C).

Combined with our docking (Figure S2), this tentatively suggests that the 1-(3-chlorophenyl)-urea moiety is important for inhibition and there is flexibility for the orientation of the benzodiazepine moiety. Since there was not much difference in the activity of the two enantiomers and the racemate of Zinc13732787, we decided to prepare and test the activity of compound **3**, a racemic mixture identical to Zinc13732787 except for an amide instead of a urea linker, which would be unable to form one of the putative hydrogen bonds described above. After five activations in the presence of 1 μM and 10 μM of **3**, currents were no different to those in the absence of compound **3**, and only after five activations in the presence of 100 μM of **3** currents were significantly smaller than in the absence of compound **3** (Figure 4D). Currents then remained significantly smaller after another five activations without any compound, reflecting slow washout. Further, intermediate 1, essentially just the benzodiazepine moiety was also included for activity assay to understand the role of appended 1-(3-chlorophenyl in Zinc13732787. However, 3-aminobenzodiazepine **1** up to 100 μM had no apparent effect on current amplitude (Figure 4D). Further, Zinc33819213, which is an acetylated version of 3-aminobenzodiazepine **1**, has shown moderate inhibition at 20 μM. Taken together, these results support the notion that Zinc13732787 engages the channel via extensive hydrogen bonding through its urea linkage.

We also tested the activity of Zinc13732787 on KCNQ1 alone. Interestingly, the compound was even more potent at reducing the current amplitude without the KCNE1 subunit; 3 μM in KCNQ1 alone yielded a similar current reduction (∼38%) compared to 20 μM in the KCNQ1/KCNE1 complex (∼48%). The V_50_, however, was not affected (Figure S9).

### Mutagenesis Analysis of Zinc13732787 Inhibition

To determine the contribution of specific amino acid residues to Zinc13732787 effects, we performed site-directed mutagenesis at key positions within the pore identified through our docking analysis. We introduced the T311S, T312S, F335Y, I337V, F340Y, and A341S mutations, which reduce hydrophobicity and subtly alter residue size, and expressed these mutant channels in *Xenopus laevis* oocytes for functional evaluation using TEVC recordings. To validate our mutagenesis approach, we first examined the effects of Chromanol 293B. Compared to wild-type (WT), T312S, I337V, and A341S KCNQ1 mutants exhibited higher remaining currents, indicating a reduced, albeit not significant unless removing outliers (Figure 5, Figure S10). These results align with our predictions and previous studies, which identified these residues as key contributors to Chromanol 293B binding.^32^ Docking analysis indicated that residues T311 and F340 might be important for Zinc13732787 binding. However, the small currents observed in the T311S and F340Y mutants made precise measurements challenging, leading us to exclude the data from these mutants in our analysis of inhibitor sensitivity. These small currents may be due to the mutations either abolishing channel function or preventing the channel from being properly expressed on the oocyte surface. Next, we assessed the impact of these mutations on Zinc13732787 inhibition. At 20 μM, the I337V and A341S mutants showed higher remaining current than WT, suggesting reduced inhibition. However, these differences were not statistically significant (*p* > 0.05, one-way ANOVA with posthoc Dunnett’s test). Unexpectedly, T312S did not alter ligand inhibition, despite its proximity to Zinc13732787 in our docking analysis. However, serine residues in mutant T312S channels retain the same putative hydrogen bonds as WT with Zinc13732787 predicted by our docking, perhaps explaining the inhibition of the mutant by Zinc13732787 and highlighting a subtle difference between the way these threonine residues engage the different drugs.

**Figure 5:**
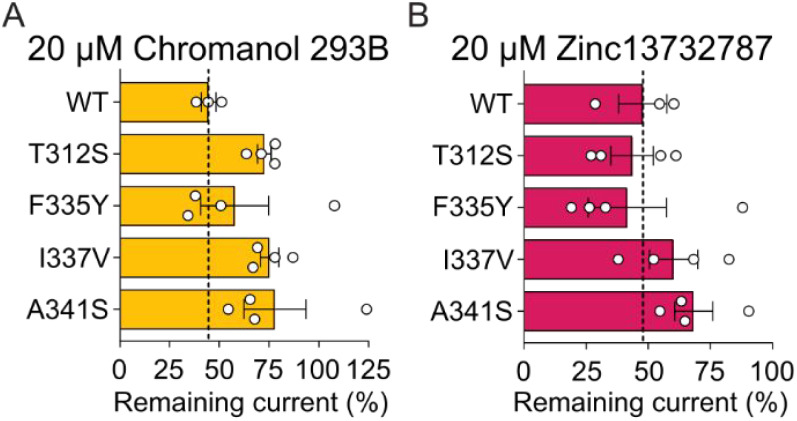
Impact of pore mutations on drug modulation of KCNQ1/KCNE1 currents. Averaged and normalized K^+^ currents through mutant KCNQ1/KCNE1 channels activated by +40 mV were measured in the presence of 20 μM Chromanol 293B (yellow) or Zinc13732787 (magenta). Using the full data set, a one-way ANOVA showed no significant difference between the mutant channels treated with either compound and the WT (*F* = 1.28 for Chromanol 293B; *F* = 1.02 for Zinc13732787; *n* = 1–4).

Overall, these findings suggest that residues Ile337 and Ala341 may contribute to the binding of Zinc13732787, likely through hydrophobic interactions. The lack of significant changes in Zinc13732787-treated mutants implies that additional residues or cooperative interactions may play a role in binding. Further mutational and structural studies are needed to better define the molecular determinants of inhibitor binding.

### Lack of Effect of Zinc13732787 on the KCNQ2/KCNQ3 Channel

To further characterize the inhibitory potential of Zinc13732787 on the related KCNQ2/KCNQ3 channels (the main contributor to the neuronal M-current^38^ ), we examined its effect on human KCNQ2/KCNQ3 channels expressed in *Xenopus laevis* oocytes using TEVC. Interestingly, neither 10 or 100 μM of Zinc13732787 showed effects on V_50_, maximal conductance (*G*_max_), or current amplitude at −40 mV of KCNQ2/KCNQ3 (Figure 6). Hence, our results show that at concentrations clearly inhibiting KCNQ1/KCNE1, there were no effects of Zinc13732787 on KCNQ2/KCNQ3.

**Figure 6:**
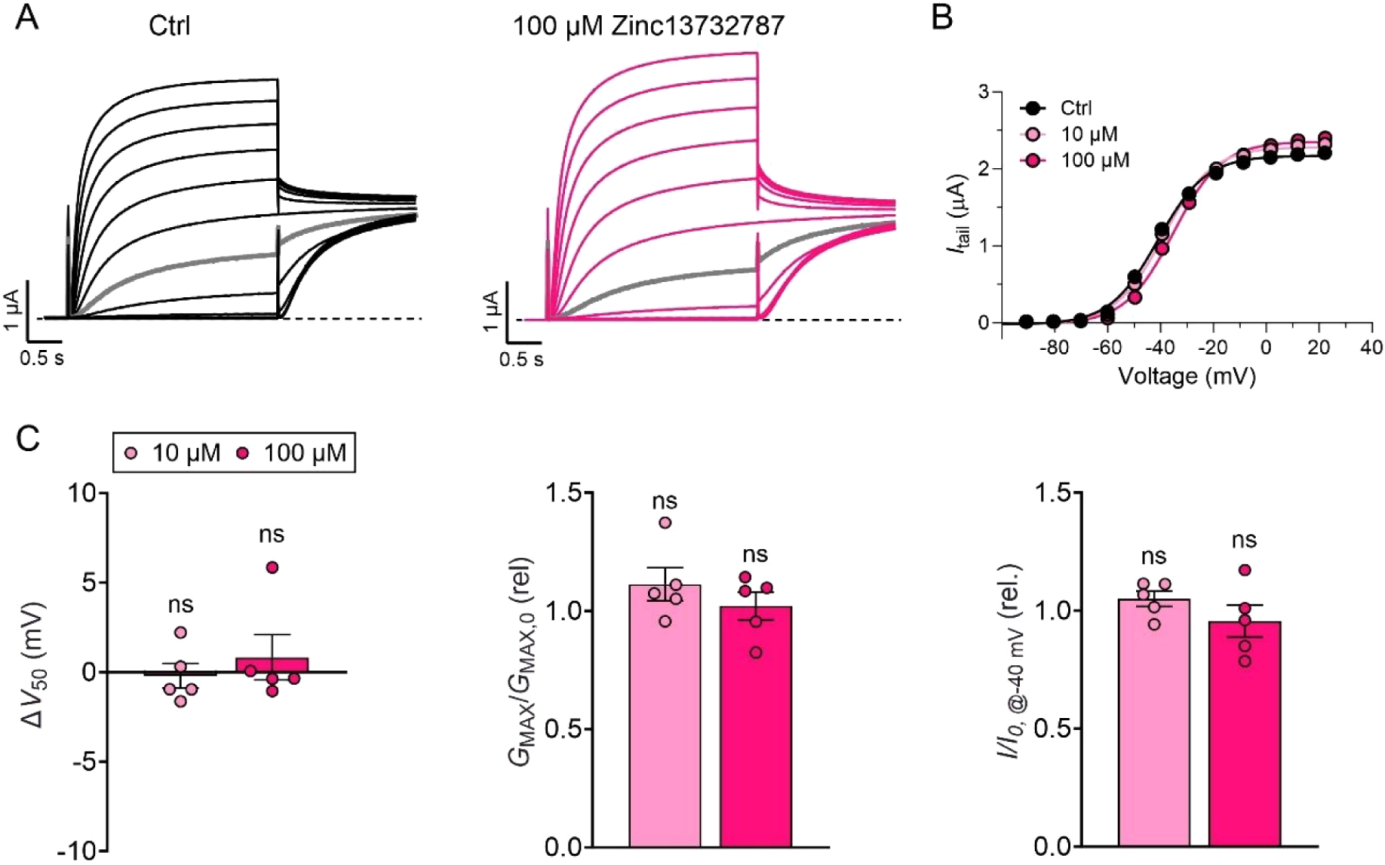
Lack of effect of Zinc13732787 on KCNQ2/KCNQ3 channels. A, Example recording of KCNQ2/KCNQ3 under control conditions (black traces) and after application of 100 μM (pink traces) in the presence of the indicated concentration of Zinc13732787. Currents were generated from a holding voltage of −80 mV, followed by a prepulse to −100 mV for 2 s, and activating voltage steps from −100 to +20 mV in 10 mV increments for 3 s each. The tail voltage was −30 mV. The gray trace corresponds to the current generated at an activation step to −40 mV. B, Corresponding conductance versus voltage curve for the example shown in A, with also 10 μM included (light pink). Curves in B represent Boltzmann fits. C, Mean effect of indicated concentrations of Zinc13732787 on KCNQ2/KCNQ3 V_50_ (left), *G*_max_ (middle), and current amplitude at −40 mV (right). Data shown as mean ± SEM. Statistics denote one-way ANOVA followed by Šídák’s multiple comparisons test. ns denotes *p* > 0.05. *n* = 5.

### Effects of Zinc13732787 on Cell Proliferation, Cytotoxicity, and Neurite Outgrowth in iPSC-Derived Neuronal Stem Cells

Genetic variants in *KCNQ1* are associated with both peripheral tissue and CNS disorders, including long QT syndrome, diabetes, and epilepsy.^39,40^ Emerging evidence from human and preclinical studies suggests that *KCNQ1*, and its encoded channel Kv7.1, may play a critical role in neurological dysfunction and represent a potential therapeutic target for epilepsy and cognitive impairments.^39–42^ Previous work demonstrated that the loss of KCNQ1 and pharmacological inhibition with the antagonist JNJ303 reduce neurite outgrowth in induced pluripotent stem cell-derived neural stem cells (NSCs) during early neuronal differentiation. This points to a role for KCNQ1 in early neuronal development.^26^

To test if Zinc13732787 as a KCNQ1 antagonist also impacts neurite outgrowth, we tested this compound in the same cell lines used in the previous study. Human iPSC lines with a *KCNQ1*-knockout (KO) were generated^26^ by deleting exons 4 and 5 of KCNQ1 (hg38 MANE transcript NM_000218.3). One WT control and one isogenic homozygous *KCNQ1-KO* cell line were selected and differentiated into NSCs.

First, we assessed if treatment with the putative KCNQ1 antagonist Zinc13732787 affects cell proliferation and causes cytotoxic effects in NSCs. Therefore, the WT control line was treated with different concentrations of Zinc13732787 (1, 5, 10, 25, and 50 μM). None of these treatments seemed to affect NSC proliferation (Figure 7A,B) and did not reveal cytotoxic effects when tested up to a 50 μM final concentration (Figure 7C).

**Figure 7:**
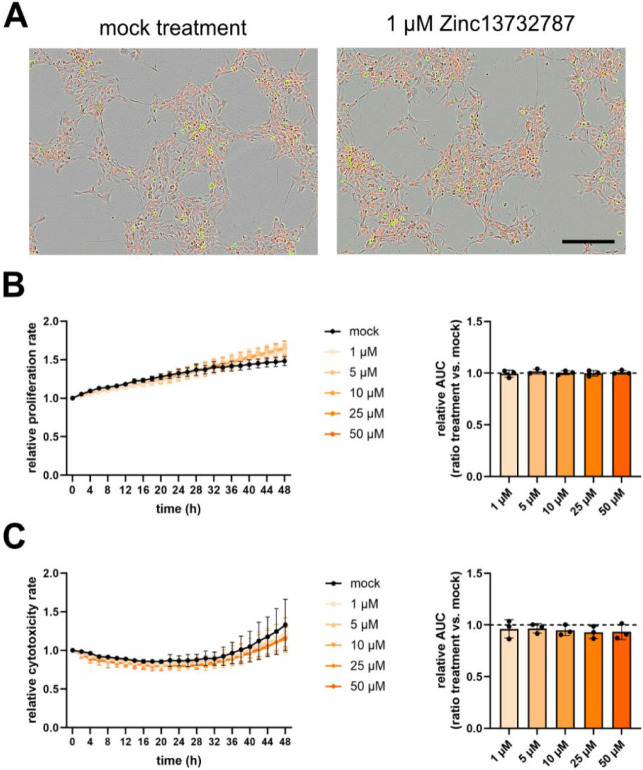
Proliferation and cytotoxicity rate of WT cells following treatment with Zinc13732787. WT NSCs were treated with different concentrations of Zinc13732787 and compared to mock treatment (DMSO only). A, Representative pictures from the Incucyte S3 live cell analysis instrument of cells expressing Nuclight Rapid Red and Cytotox Green dyes after 48 h. Scale bar 200 μm. B, Proliferation analysis. Right side: The area under the curve (AUC) was calculated and analyzed by a one-way ANOVA with correction for multiple testing according to the Bonferroni method. Left side: Time response was analyzed by using a two-way ANOVA (time × treatment), *n* = 3. C, Cytotoxicity analysis. Right side: The AUC was calculated and analyzed by a one-way ANOVA with correction for multiple testing according to the Bonferroni method. Left side: Time response was analyzed by using a two-way ANOVA (time × treatment, *n* = 3).

Then, we tested the impact of different Zinc13732787 concentrations on neurite outgrowth (Figure 8A,B). Treatment of WT NSCs with low concentrations of Zinc13732787 (1, 5, and 10 μM) revealed a significant reduction of neurite length, whereas high concentrations of Zinc13732787 (25 and 50 μM) showed no alteration in neurite length. Taken together, these results indicate that the newly identified KCNQ1 antagonist Zinc13732787 reduced neurite length when used at low concentrations, which is consistent with the results for the known KCNQ1 antagonist JNJ303.^26^

**Figure 8:**
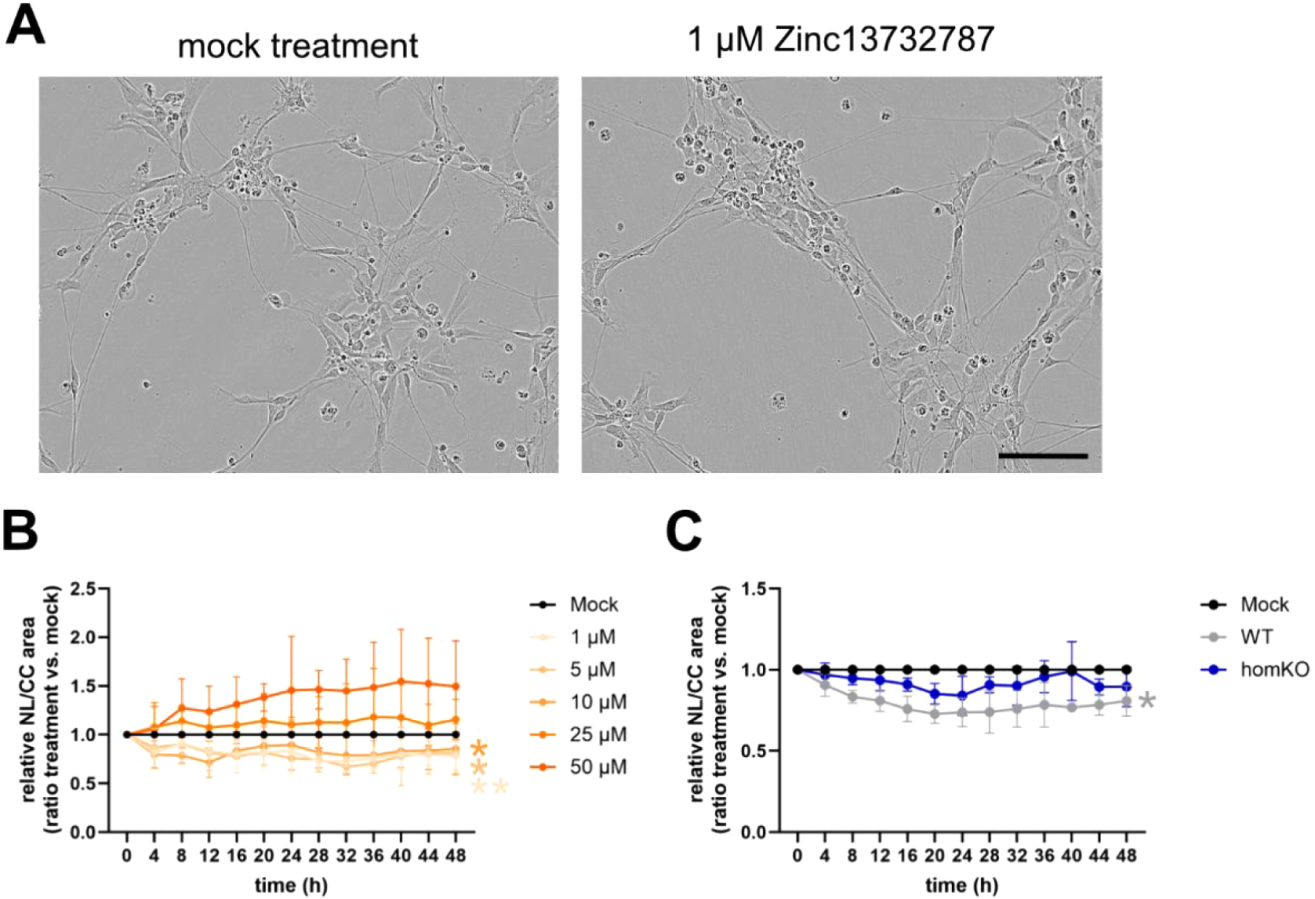
Assessment of neurite outgrowth during early neuronal differentiation following treatment with Zinc13732787. Neurite outgrowth was measured during the first 48 h after treatment with the Incucyte instrument. Neurite length (NL) was normalized to cell body cluster (CC) area. Each treatment was compared to mock treatment (DMSO only). A, Representative images of cells after 48 h of plating. Scale bar 100 μm. B, WT NSCs were treated with different concentrations of Zinc13732787 Two-way ANOVA. C, WT and homozygous *KCNQ1*-KO, NSCs were treated with 1 μM Zinc13732787 and mock (DMSO), and neurite length was measured for 48 h. The graph presents the normalized neurite length per cell body cluster area relative to the corresponding mock treatment. Two-way ANOVA, *p < 0.05, **p < 0.01, *n* = 4.

To assess whether the effect of Zinc13732787 on neurite outgrowth is specifically mediated by KCNQ1, we compared the responses of homozygous *KCNQ1*-KO and WT NSCs. Treatment with 1 μM Zinc13732787 significantly reduced neurite outgrowth in differentiating WT NSCs, whereas no effect was observed in *KCNQ1*-*KO* cells. The absence of a response in *KCNQ1-KO* cells indicates that the reduction in neurite outgrowth is specifically dependent on KCNQ1 function, suggesting that Zinc13732787 acts directly on KCNQ1. These findings are consistent with previous results demonstrating that both genetic ablation and pharmacological inhibition of KCNQ1 reduce neurite outgrowth during early neuronal differentiation.

### Zinc13732787 Binding Mode Analysis

We used the RosettaLigand program^43,44^ to predict the binding mode of Zinc13732787 and compare it with our existing prediction results for this ligand. RosettaLigand generated 200 conformations by applying the BCL:CONF algorithm.^45^ Analysis of the 10 poses with the lowest energy minima revealed that in 6 out of 10 poses, the Zinc13732787 binding modes were similar to those predicted by Glide and were surrounded by key interacting residues Thr311–312, Ile337, Phe340, and Ala341 (Figure 9, left panel). Next, we explored how Zinc13732787 interacts with the open (PDB ID 6V01) and closed (PDB ID 6UZZ) forms of KCNQ1. Our docking results showed that the ligand Zinc13732787 is positioned just beneath the selectivity filter and occupied the same position in the closed form, albeit in a different conformation (Figure 9, right panel).

**Figure 9:**
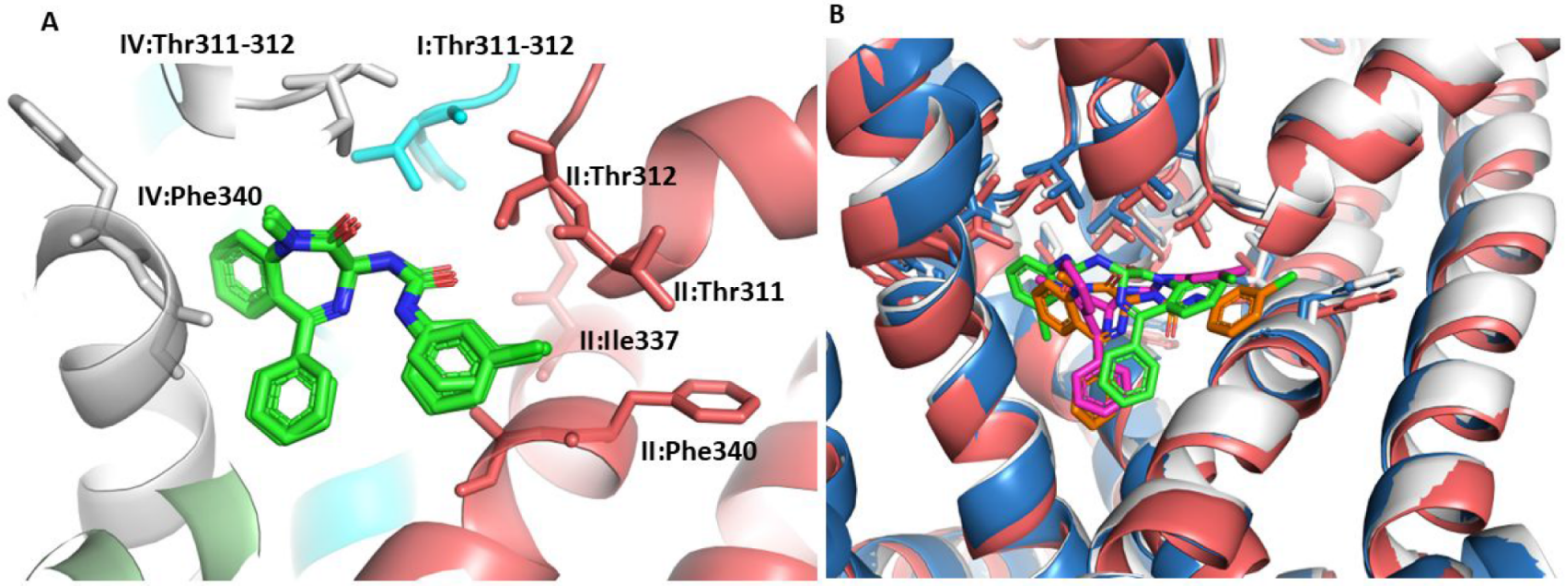
Ensemble ligand docking of Zinc13732787 and KCNQ1. RosettaLigand docking produced 10 lowest-energy binding conformations, with 6 out 10 models (60%) showing the same conformation as predicted (panel A). The right panels (panel B) compare the binding mode of Zinc13732787 with KCNQ1 in its closed (PDB ID 6UZZ), activated-closed (PDB ID 6V00) and Open (PDB ID 6V01) states. The ligand Zinc13732787 is shown as sticks, orange in the open, green in the activated-closed, and pink in the closed KCNQ1 states. The KCNQ1 structure is colored in deep salmon, gray, and sky blue for KCNQ1 open, activated-closed, and closed forms, respectively. The interacting amino acids are represented as sticks.

## Discussion

Voltage-gated potassium channel KCNQ1 plays a critical role in regulating electrical excitability in the heart, brain, and other tissues. The channel is considered an attractive target for developing new therapeutics for KCNQ1-related channelopathies. In this work, we carried out molecular docking combined with electrophysiological testing to identify small molecules that target KCNQ1. Here, we used KCNQ1 cryo-EM structures^4^ as a framework for virtual screening of chemical libraries (*n* = 36,374 compounds). Finally, 8 selected ligand hits were tested in *Xenopus laevis* oocytes. Our findings revealed that out of these 8 ligands, only the benzodiazepine-based Zinc13732787 significantly inhibits KCNQ1/KCNE1 function. The rest of the ligands showed moderate or minimal effects on the channel current. Further investigations into the cellular effects of Zinc13732787 showed no impact on proliferation or cytotoxicity in human NSCs.

The identification of Zinc13732787 as an inhibitor is particularly noteworthy given the channel’s critical role in cardiac repolarization and its association with various cardiac arrhythmias. The binding mode analysis of Zinc13732787 revealed that it interacts with key residues within the KCNQ1 channel pore, including Thr312, Ile337, and Phe340, in a manner reminiscent of the known KCNQ1 inhibitor, Chromanol 293B.^30,46,47^ However, despite the structural differences between these two compounds, Zinc13732787 appears to be a more potent inhibitor of KCNQ1/KCNE1 channel function, as evidenced by its ability to significantly reduce current amplitudes at lower concentrations compared to Chromanol 293B. Notably, the inhibitory effect of Zinc13732787 persisted even after washout, in contrast to the more reversible inhibition observed with Chromanol 293B. Further, selected analogues of Zinc13732787 were synthesized and tested against KCNQ1/KCNE1. These experiments showed that the urea group and the chlorobenzene are important for the inhibitory activity of Zinc13732787 toward KCNQ1/KCNE1.

In addition to the promising inhibitory effects of Zinc13732787 on KCNQ1/KCNE1 channel function, our *in silico* ADME and toxicity predictions suggest that this compound possesses favorable pharmacokinetic properties, including the ability to permeate the BBB, lack of hERG or cytochrome p450 enzyme inhibition, and it was not predicted to be a P-gp substrate.^34^ These characteristics further support the potential utility of Zinc13732787 as a chemical probe or primary hit compound for the development of KCNQ1-targeted therapies.

The identification of Zinc13732787 as a potent and persistent KCNQ1 inhibitor represents an important step toward understanding the functional role of this ion channel. Among the various channels tested (KCNQ1/2/3), Zinc13732787 appeared to be more potent for KCNQ1. Furthermore, low concentrations of the compound reduced neurite outgrowth during early neuronal differentiation exclusively in WT human NSCs with no effect observed in homozygous KCNQ1-KO cells. This suggests that the compound selectively targets KCNQ1 without evidence of off-target effects. Although KCNQ1 is best known for its role in cardiac repolarization, its neuronal function remains poorly characterized despite documented expression in brain tissue.^11,14^ Here, we identify Zinc13732787 as a novel KCNQ1 antagonist that recapitulates the neurite outgrowth phenotype observed in KCNQ1 knockout NSCs and after treatment with the established antagonist JNJ303, supporting a role for KCNQ1 activity during neuronal differentiation.^26^ Because the functional consequences of epilepsy-associated KCNQ1 variants in neurons remain unclear and may involve gain- or loss-of-function mechanisms,^48^ dedicated neuronal studies will be required to determine whether KCNQ1 agonists or antagonists may represent appropriate therapeutic strategies.^1^

Benzodiazepine-based drugs are widely used as antiepileptic, anxiolytic, and sedative medications. This class of drugs works primarily by modulating the GABA-A receptor, a ligand-gated ion channel in the central nervous system.^49^ To date, there is no evidence that commonly used benzodiazepines, such as diazepam or lorazepam, directly modulate KCNQ1 or other channels of the KCNQ family. However, some benzodiazepine derivatives can also bind to peripheral benzodiazepine receptor transporters or may function as blockers or activators of *I*_*Ks*_ channels (KCNQ1-KCNE1),^50,51^ thereby suggesting a broader pharmacological impact for this class of compounds.

Our study identifies Zinc13732787, a benzodiazepine-based compound, as a selective inhibitor of the KCNQ1/KCNE1 potassium channel complex, raising important questions about the potential cross-reactivity of this chemical class with other Kv channels and the implications for neurological and cardiac comorbidities. This is particularly intriguing in the context of epilepsy, where both KCNQ2/3 loss-of-function and cardiac KCNQ1 mutations have been associated with sudden unexpected death in epilepsy (SUDEP).^11,52^ Moreover, the comorbidity of cardiac arrhythmias and neurological conditions like epilepsy and anxiety disorders suggests a shared molecular etiology involving ion channels such as KCNQ1. Given the emerging view of KCNQ1’s role in brain development and function, compounds like Zinc13732787 could serve as valuable tools to explore how potassium homeostasis in both the heart and brain contributes to disease phenotypes.

However, further investigations are necessary to fully elucidate the therapeutic potential of Zinc13732787, especially through *in vivo* validation in mammalian systems, specificity testing against additional KCNQ isoforms, as well as structural studies of the KCNQ1-Zinc13732787 complex to better understand the interaction and stability of the complex at the molecular level. Differentiation of human WT and isogenic *KCNQ1-KO* human-induced pluripotent stem cells (iPSCs) into cardiomyocytes, neurons, glial cells, and heart and brain organoids will enable functional investigation of the compound in more complex human cell models, thereby enhancing our understanding of its specificity and cellular responses.

While these results are promising, there were limitations that should be acknowledged. First, pharmacokinetic properties were derived from *in silico* prediction tools and not validated through experimental pharmacokinetic or safety studies. Second, our virtual screening was performed on a KCNQ1 structure in complex with KCNE3, whereas electrophysiological testing was conducted on the KCNQ1/KCNE1 complex in oocytes. Although these auxiliary subunits share overlapping binding interfaces with the pore-forming α-subunit, structural differences between KCNE isoforms may impact ligand accessibility and conformation. Considering limited experimental resources, we prioritized compound selection based on predicted affinity and chemical diversity, which may have restricted the discovery of additional functional hits. The consistent neurite outgrowth phenotype observed after KCNQ1 genetic deletion and pharmacological inhibition supports a role for KCNQ1 activity in neuronal differentiation. However, direct electrophysiological evidence for Kv7 current modulation in human neural stem cells is currently lacking. Future studies examining KCNQ1-dependent ion permeability and slow-inactivating Kv7 currents will be important to further define the mechanisms linking channel activity to neurite development.

## Conclusion

In this study, we carried out a high-throughput virtual screening followed by electrophysiological testing, which identified several ligands with promising characteristics. Of the 36,374 compounds screened, 8 ligands were prioritized based on predicted binding affinity, predicted pharmacokinetic properties, and chemical diversity. Functional validation in *Xenopus laevis* oocytes revealed that Zinc13732787, a benzodiazepine-derived compound, selectively and potently reduced the current amplitude of KCNQ1/KCNE1 channels but showed no effects on KCNQ2/KCNQ3 channels. Further, the activity profile of selected analogues against KCNQ1/KCNE1 showed that the 3-(3-chlorophenyl) urea substituent is required for the inhibition. Our docking results highlighted that the ligands bind beneath the KCNQ1 pore and interact with the selectivity filter, consistent with findings from studies of the known inhibitor Chromanol 293B. In human iPSC-derived NSCs, Zinc13732787 acted as a KCNQ1 inhibitor, demonstrating both functional blockade and high specificity. In summary, our results establish Zinc13732787 as a promising lead compound for dissecting the physiological and pathological roles of KCNQ1. Given the channel’s involvement in cardiac repolarization and emerging roles in the brain, this compound offers a valuable starting point for further exploration of heart-brain comorbidities and for the development of targeted therapies for KCNQ1-related disorders.

## Materials and Methods

### Virtual Screening

#### KCNQ1 Binding Site for Docking

Our study utilized human KCNQ1 cryo-EM structures (PDB IDs 6V00, 6V01, and 6UZZ). We employed the programs P2Rank,^27^ FTSite,^28^ and Site-map^29^ to identify potential small-molecule binding sites on KCNQ1. Although the KCNQ1 structure and function are modulated by lipids, ligands, and partner molecules with various interaction sites, we focused specifically on the pore region and binding sites near the S5 and S6 segments. In addition, we docked and analyzed the binding mode and interacting amino acids in KCNQ1 of chromanol 293B, a known KCNQ1 inhibitor.^30,32^

#### Protein Preparation

The cryo-EM structures of human KCNQ1 (PDB IDs 6V00, 6V01, and 6UZZ, Uniport ID: P51787) were retrieved from the protein data bank (www.rcsb.org) and prepared using the protein preparation workflow (Maestro, Schrödinger) for virtual screening. The missing side chains and loops were modeled using Prime,^53^ and metal ions, cofactors, and water molecules were removed. Finally, the structure (PDB ID 6V00) was optimized using the OPLS5 force field for molecular docking.^54^

#### Receptor Grid Generation

The receptor grid for the KCNQ1-apo structure (PDB ID 6V00) was generated using the Chromanol 293B binding mode^30,32^ and predicted binding site information obtained from P2Rank,^27^ FTSite,^28^ and Site-map,^29^ with the centroid defined by selected residues Val310, Thr311–312, Ile337, Phe340, and Ala341 using the Site in Glide. A cubic box with an edge length of 17 Å (default grid spacing) was used for ligands to be docked.

#### Chemical Library Preparation

We prepared a chemical library compiling Prestwick chemical library of FDA-approved drugs (PCL, *n* = 1280, https://www.prestwickchemical.com/), Neuronal signaling library (NSL, *n* = 556), and Zinc *in vivo* subsets (*n* = 27,338) (zinc20.docking.org).^33^ Additionally, we included known KCNQ1 ligands from the literature and utilized Swiss Drug Design tools^55^ to generate a targeted (KCNQ1-targeted) library (*n* = 7,200) using the ECP4 fingerprint.^31^ We included the PCL, NSL, and ZINC *in vivo* subsets in our screening for their distinct advantages: PCL comprised FDA-approved drugs, ensuring compound safety and bioavailability for drug repurposing. NSL contained small molecules that modulate neuronal signaling pathways. ZINC *in vivo* subsets offered compounds with *in vivo* test results and favorable pharmacokinetic properties, increasing the likelihood of a successful screening outcome. The ligands were retrieved and processed for library generation. The chemical libraries were prepared for docking by optimizing the compound using the OPLS_2005 force field.^56^ The retrieved compounds were filtered by following Lipinski’s “Rule of 5”.^57^ The Epik classic program was applied to generate protonation states at a target pH 7.00 (±2).^58^ During ligand preparation, the ligands were kept in their specified chiral forms. Additionally, any salts were removed, and different tautomeric forms of the compounds were generated using LigPrep from Schrödinger.^59,60^

#### Molecular Docking

Molecular docking was performed by using the High-Throughput Virtual Screening (HTVS) workflow in Glide (Maestro, Schrödinger Release 2020-3). The 3,637 top-scoring hits from HTVS were redocked with the more thorough SP scoring function in Glide. After visual inspection and alignment of receptor conformations, the most promising binding poses were selected for further analysis with MM-GBSA.

#### Prime MM-GBSA

The selected (*n* = 363) top hits from the initial screening against KCNQ1-apo were further used to calculate binding free energy using Prime MMGBSA (v3.000). The program used the molecular mechanics generalized Born surface area (MM-GBSA) protocol with a novel energy model, VSGB, as the solvation model.^61^ The binding poses of the receptor–ligand complexes were minimized by Prime and the binding energies of the different poses were predicted by applying the OPLS-2005 force field. The following formula was used to calculate the binding free energy:

1 ΔG(bind)=ΔG(solv)+ΔE(MM)+ΔG(SA)

where Δ*G*(solv) is the change in solvation free energy, calculated as the GBSA solvation energy of the protein–ligand complex minus the sum of the solvation energies of the unbound protein and ligand. Δ*E*(MM) is the difference between the minimized energy of the protein–ligand complex and the sum of the energies of the unbound protein and ligand. Δ*G*(SA) is the change in surface area-dependent energy, representing the nonpolar contribution to solvation, computed as the surface area energy of the complex minus the sum of the surface area energies of the unbound protein and ligand.

#### *In Silico* Prediction of Compounds Toxicity, Pharmacokinetic Properties (PK), and ADMET Parameters

The 136 compounds selected from MM-GBSA were further evaluated for pharmacological feature selection and toxicity prediction. We carried out an assessment analysis of the selected hits for ADMET properties. Additionally, pharmacological features like BBB permeability, P-gp substrate binding, Pan-assay interference compounds (PAINS) filtration, and cardiac cytotoxicity were predicted using SwissADME (http://www.swissadme.ch/) and pkCSM (https://biosig.lab.uq.edu.au/pkcsm/) web-based servers.^35^ SwissADME applies multiple parameters to assess a ligand’s ability to cross the BBB and gastrointestinal (GI) absorption. The program also predicts interactions with P-gp, cytochromes P450 (CYP1A2) enzymes, and the human Ether-à-go-go-Related Gene (hERG) potassium ion channel.

### Heterologous Expression of KCNQ1/KCNE1 in *Xenopus laevis* Oocytes

Prof. Stephan Pless (University of Copenhagen) provided human KCNQ1 and KCNE1 cDNAs, codon optimized for *Homo sapiens*, inserted into a customized pCDNA3.1­(+) vector containing *Xenopus* globin 3′UTR­(Untranslated region) and a polyadenylation signal. To introduce specific mutations, we employed recombinant PCR with Phusion High-Fidelity DNA polymerase (catalog number F-54L, Thermo Fisher Scientific), adopting a method detailed elsewhere.^62^ The plasmids were confirmed using Sanger DNA sequencing (Genewiz, Germany), linearized using NotI, and cRNA was produced *in vitro* using T7 Polymerase (mMessage mMachine kit, Ambion). 6 ng (KCNQ1) and 2.4 ng (KCNE1) of cRNA were coinjected into stage V/VI *Xenopus laevis* oocytes purchased from Ecocyte Bioscience (Dortmund, Germany). The oocytes were cultured at 18 °C in 50% Leibovitz medium (Merck) supplemented with 0.25 mg/mL gentamicin, 1 mM l-glutamine, and 15 mM HEPES (pH 7.6). cRNA for human KCNQ2 and KCNQ3 was prepared as previously described.^63^ 2.5 ng cRNA of KCNQ2 and 2.5 ng cRNA of KCNQ3 were coinjected into each oocyte and stored at 16 °C for 2 days before experiments.

### Electrophysiological Recording and Analysis

One day after injection, currents were recorded using TEVC employing an OC-725C amplifier (Warner Instruments) and LIH 8 + 8 digitizer with Patch master software (HEKA), at 20 kHz frequency, filtered at 100 Hz. A micropipette puller, model PC-100 (Narishige, Japan), was used to pull recording microelectrodes with resistances ranging from 0.5 to 1.5 MΩ from borosilicate glass capillaries (Harvard Apparatus, USA). Oocytes were clamped at −80 mV and continuously perfused with a bath solution containing (in mM): 96 NaCl, 2 KCl, 1.8 CaCl_2_, 1 MgCl_2_, 5 HEPES, and 0.2% DMSO; pH 7.4 with NaOH. The different compounds were solubilized in DMSO and then diluted to 20 μM in bath solution, unless otherwise indicated. Depolarizing voltage pulses were applied for 6 s, ranging from −40 to +90 mV in 10 mV increments, and then stepped back to +20 mV for 1 s to determine the voltage dependence of channel activation. For selected compounds (i.e., Chromanol 293B and Zinc13732787), maximal current amplitude was determined in five consecutive activations at currents activated by steps to +40 mV without compound (resting 10 s between activations), followed by five activations in increasing compound concentrations (1, 10, and 100 μM), and then five activations without compound as a washout. For experiments on KCNQ2/KCNQ3, corresponding experiments were performed using a Dagan CA-1B amplifier (Dagan), with currents sampled at 5 kHz and filtered at 500 Hz. The holding voltage was −80 mV. Following a prepulse to −100 mV for 2 s, depolarizing voltage pulses were applied for 3 s, ranging from −100 to +30 mV in 10 mV increments, and then stepped back to −30 mV for 2 s to determine the voltage dependence of channel activation. The sweep-to-sweep interval was 30 s. For KCNQ2/KCNQ3, either control solution or compound-supplemented control solution was added continuously using a peristaltic pump (0.5 mL/min, ISM597D, Labinett Lab AB) for a minimum of 5 min.

Following the acquisition of the current amplitude from pClamp, Prism v10 (GraphPad Software) was used for all data analysis. Half-maximal voltage (V_50_) for each compound was calculated by plotting the tail current amplitudes at +20 mV for each cell and fitting them with a Boltzmann sigmoidal function. Remaining current amplitude after drug application was measured at currents activated by steps to +40 mV and normalized to currents activated by steps to +40 mV in the absence of the drug. KCNQ1/KCNE1 currents activated by +40 mV were fitted with a single exponential function to establish activation time constants (τ + 40). For all measurements, a one-way ANOVA followed by a post hoc Dunnet’s or Tukey’s HSD test was used to determine statistical differences. To highlight the most efficacious compound, only comparisons with statistical significance (*p* < 0.01) are marked with an asterisk. For KCNQ2/KCNQ3, the effect on half-maximal voltage (V_50_) and maximal conductance (*G*_max_) was calculated from the plotted tail current amplitudes at −30 mV as a function of the preceding test voltage for each cell under control conditions and in the presence of the compound and fitting them with a Boltzmann sigmoidal function. The effect on current amplitude at −40 mV was calculated from the steady-state current at the end of the activating current trace at −40 mV. For the KCNQ2/KCNQ3 measurements, a one-way ANOVA followed by Šídák’s multiple comparisons test was used to determine statistical differences.

## Cell Culture

### Generation of Cell Lines

Human-induced pluripotent stem cells (iPSCs) with a *KCNQ1* knockout (KO) were generated in a previous study (unpublished data). A homozygous *KCNQ1* knockout (KO) iPSC line and an isogenic control wild-type (WT) iPSC line were selected for the study. The cell lines were induced into neuronal stem cells (NSCs) according to Yan et al.^64^ After induction, NSCs were cultured in neuronal expansion medium (NEM) (Neurobasal (A1647801, Thermo Fisher), Advanced DMEM/F12 (12634010, Thermo Fisher), PenStrep (15140122, Thermo Fisher), Neural Induction Supplement (A1647801, Thermo Fisher)), split every 4–5 days when reaching 100% confluency and used for experiments between passages P6 to P10.

### Proliferation and Cytotoxicity Experiment

To measure proliferation and cytotoxicity, NSCs were split with Accutase (A1110501, Thermo Fisher), and 25,000 cells were seeded on Geltrex (A1413302, Thermo Fisher) coating into a 96-well format in NEM. After 16 h, cells were simultaneously treated with two different dyes: NucLight Rapid Red (4717, Sartorius) to assess proliferation and Cytotox Green Dye (4633, Sartorius) to evaluate cytotoxicity. Additionally, cells were either treated with Zinc13732787 or DMSO (mock). After treatment, cells were immediately placed into the IncuCyte S3 Live-Cell Analysis Instrument (Sartorius), and the first recording was started within 30 min. Cells were recorded every 2 h for 48 h.

### Neurite Outgrowth Experiment

To measure neurite outgrowth, NSCs were grown in maturation medium (MM) (Neurobasal medium, Advanced DMEM/F12, Glutamax (35050061, Thermo Fisher), N2 (17502048, Thermo Fisher), B27 (17504044, Thermo Fisher), PenStrep) for 3 days prior to the experiment. NSCs were split with Accutase, and 23,000 cells were seeded on ornithine (P4957, Sigma-Aldrich) and laminin (L2020, Sigma-Aldrich) coating into a 96-well format in MM. Cells were treated with either Zinc13732787 or DMSO (mock) during seeding and directly placed into the IncuCyte system. The first recording was started after 1 h, and cells were recorded every 4 h for 48 h. Neurite outgrowth was analyzed with the NeuroTracker software and normalized to the cell body cluster area by the IncuCyte system.

### Analysis

The green signal for cytotoxicity was normalized to the number of cells, determined with the Nuclight Rapid Red dye to calculate the cytotoxicity rate. To analyze proliferation, cytotoxicity rate, and neurite outgrowth, all values were first normalized to the baseline at time point 0, which was set to 1. Subsequently, each treatment time point was normalized to the corresponding time point of the mock treatment, also set to 1.

Afterward, two different analyses were carried out: 1) the time response was analyzed by a two-way ANOVA considering the two factors time and treatment; 2) the area under the curve (AUC) was calculated and analyzed by a one-way ANOVA with correction for multiple testing according to the Bonferroni method.

## Compound Synthesis

### General Information

Racemic **1**, **(** ***R*** **)-1** and **(** ***S*** **)-1** were purchased from Angene International Limited (Nanjing, China 210061). All other reagents were purchased from Sigma-Aldrich, Aaron Chemicals, or Fluorochem and were used without purification. Solvents were used as received unless otherwise stated. Thin-layer chromatography was performed on aluminum sheets coated with Merck TLC silica gel 60 F254. Visualization was accomplished by UV irradiation at 254 and 220 nm. Flash column chromatography was performed using a PuriFlash XS520Plus equipped with prepacked columns (particle size 0.040–0.063 mm, 230–400 mesh) from Interchim. The purity of all the final compounds was confirmed to be >95% (HPLC). Analytical HPLC analyses were performed using an Agilent 1260 HPLC equipped with an XBridge C18 3.5 mM column eluting with mixtures of water and acetonitrile (both containing 0.1% formic acid). ^1^H NMR spectra were recorded on a Bruker spectrometer at 600 MHz. Chemical shifts (δ) are given in ppm downfield from TMS as the internal standard. Coupling constants are given in Hertz (Hz). LC-HRMS was recorded using a Thermo Scientific Orbitrap exploris 120 with a heated ESI probe, a Thermo Scientific Vanquish UHPLC system with a UV detector, and a column: Thermo Scientific Accucore Vanquish C18+ 50 × 2.1 mm Particle size 1.5 μ. MS was recorded in ESI positive mode using a flow rate of 0.5 mL/min. Synthesis details and compound characterization data are supplied in the Supporting Information (Figures S11–S27).

### Synthesis Protocols

#### (*R*/*S*)-1-(3-Chlorophenyl)-3-(1-methyl-2-oxo-5-phenyl-2,3-dihydro-1*H*-benzo­[*e*]­[1,4]­diazepin-3-yl)­urea (**2**)

To a stirred solution of racemic 3-amino-1-methyl-5-phenyl-1,3-dihydro-2H-benzo­[*e*]­[1,4]­diazepin-2-one (50 mg, 0.188 mmol, 1.0 equiv) in anhydrous THF (3 mL) was added 1-chloro-3-isocyanatobenzene (29 mg, 0.188 mmol, 1.0 equiv). The resulting mixture was stirred for 16 h at room temperature and the progress of the reaction was monitored using TLC. After completion, THF was evaporated and without any workup, the solution was purified by flash chromatography DLE-F0012 dry load eluting with MeOH/DCM (0.5:99.5) to give the title compound (54 mg, 69%) as an off white solid. ^1^H NMR (600 MHz, (CD_3_)_2_SO): δ = 9.29 (s, 1H), 7.75 (ddd, *J* = 8.7, 7.0, 1.8, 1H), 7.70–7.65 (m, 2H), 7.57–7.52 (m, 4H), 7.48–7.45 (m, 2H), 7.37 (m, 2H), 7.27 (t, *J* = 8.1, 1H), 7.17 (ddd, *J* = 8.3, 2.1, 1.0, 1H), 6.98 (ddd, *J* = 7.9, 2.1, 0.9, 1H), 5.24 (d, *J* = 8.2, 1H), 3.41 (s, 3H); ^13^C NMR (151 MHz, (CD_3_)_2_SO): δ = 168.0, 166.5, 154.6, 143.2, 142.0, 138.1, 133.7, 132.6, 131.2, 130.9, 130.0, 129.8, 128.9, 128.6, 125.1, 122.8, 121.6, 117.5, 116.5, 68.4, 35.3; LC-HRMS (ESI): *m*/*z* calc. for C_23_H_20_ClN_4_O_2_^+^, 419.1270; found 419.1271; HPLC purity: >95% (UV 254 and 280 nm).

#### (*R*)-1-(3-Chlorophenyl)-3-(1-methyl-2-oxo-5-phenyl-2,3-dihydro-1*H*-benzo­[*e*]­[1,4]­diazepin-3-yl)­urea [(*R*)-2: Zinc13732787]

(*R*)-3-Amino-1-methyl-5-phenyl-1,3-dihydro-2*H*-benzo­[*e*]­[1,4]­diazepin-2-one (50 mg, 0.188 mmol) was treated as described in the protocol for compound **2**, to give the title compound (48 mg, 61%) as an off white solid. ^1^H NMR (600 MHz, (CD_3_)_2_SO): δ = 9.29 (s, 1H), 7.75 (ddd, *J* = 8.6, 7.0, 1.8, 1H), 7.69–7.65 (m, 2H), 7.58–7.52 (m, 4H), 7.47–7.45 (m, 2H), 7.39–7.34 (m, 2H), 7.27 (t, *J* = 8.1, 1H), 7.17 (ddd, *J* = 8.3, 2.1, 1.0, 1H), 6.98 (ddd, *J* = 8.0, 2.2, 1.0, 1H), 5.24 (d, *J* = 8.2, 1H), 3.42 (s, 3H); ^13^C NMR (151 MHz, (CD_3_)_2_SO): δ = 168.0, 166.5, 154.6, 143.2, 142.0, 138.1, 133.7, 132.6, 131.2, 130.9, 130.0, 129.8, 128.9, 128.7, 125.1, 122.8, 121.6, 117.5, 116.5, 68.4, 35.3; LC-HRMS (ESI): *m*/*z* calc. for C_23_H_20_ClN_4_O_2_^+^, 419.1270; found 419.1271; HPLC purity: >95% (UV 254 and 280 nm).

#### (*S*)-1-(3-Chlorophenyl)-3-(1-methyl-2-oxo-5-phenyl-2,3-dihydro-1*H*-benzo­[*e*]­[1,4]­diazepin-3-yl)­urea (*S*)-2

(*S*)-3-Amino-1-methyl-5-phenyl-1,3-dihydro-2*H*-benzo­[*e*]­[1,4]­diazepin-2-one (50 mg, 0.188 mmol) was treated as described in the protocol for compound **2**, to give the title compound (47 mg, 60%) as an off white solid. ^1^H NMR (600 MHz, CDCl_3_): δ = 7.65–7.61 (m, 4H), 7.49–7.43 (m, 3H), 7.40–7.36 (m, 3H), 7.26–7.27 (m, 1H), 7.19–7.15 (m, 2H), 7.12 (t, *J* = 8.0, 1H), 6.96 (ddd, *J* = 7.9, 2.1, 1.0, 1H), 5.61 (d, *J* = 8.1, 1H), 3.53 (s, 3H); ^13^C NMR (151 MHz, CDCl_3_): δ = 168.9, 167.4, 154.5, 142.7, 140.2, 137.9, 134.5, 132.0, 130.7, 130.6, 129.9, 129.8, 129.2, 128.3, 124.8, 122.9, 121.7, 119.7, 117.6, 68.5, 35.6; LC-HRMS (ESI): *m*/*z* calc. for C_23_H_20_ClN_4_O_2_^+^, 419.1270; found 419.1271; HPLC purity: > 95% (UV 254 and 280 nm).

#### (*R*/*S*)-2-(3-Chlorophenyl)-*N*-(1-methyl-2-oxo-5-phenyl-2,3-dihydro-1*H*-benzo­[*e*]­[1,4]­diazepin-3-yl)­acetamide (**3**)

To a stirred solution of racemic 3-amino-1-methyl-5-phenyl-1,3-dihydro-2*H*-benzo­[*e*]­[1,4]­diazepin-2-one (50 mg, 0.188 mmol) and 2-(3-chlorophenyl)­acetic acid (35 mg, 0.207 mmol) in DMF (5 mL) was added 1-ethyl-3-(3-dimethylaminopropyl)­carbodiimide (54 mg, 0.282 mmol) and 1-hydroxybenzotriazole (43 mg, 0.282 mmol) followed by triethylamine (65 mL, 0.376 mmol). The reaction mixture was stirred for 12 h at room temperature and the progress of the reaction was monitored using TLC. After completion, a saturated solution of NaCl was added to the reaction mixture and extracted with ethyl acetate (3×). The combined organic layers were dried over MgSO_4_ and evaporated under reduced pressure. The crude mixture was loaded onto silica (230–400 mesh particle size) and purified on a PF-DLE-F0012 column eluting with a MeOH/DCM gradient. Pure fractions were collected, evaporated, and recrystallized using DCM: Pet Ether to give the title compound (62 mg, 79%) as an off white solid. ^1^H NMR (600 MHz, CDCl_3_): δ = 7.61 (td, *J* = 6.9, 1.7, 3H), 7.50–7.47 (m, 1H), 7.42–7.36 (m, 6H), 7.33–7.29 (m, 2H), 7.27–7.24 (m, 1H), 5.53 (d, *J* = 7.9, 1H), 3.71 (s, 2H), 3.48 (s, 3H); ^13^C NMR (151 MHz, CDCl_3_): δ = 170.2, 167.7, 167.6, 142.7, 138.0, 136.4, 134.6, 132.0, 130.8, 130.7, 130.0, 129.8, 129.7, 129.1, 128.3, 127.7, 127.5, 124.6, 121.6, 67.3, 43.2, 35.4; LC-HRMS (ESI): *m*/*z* calc. for C_24_H_21_ClN_3_O_2_^+^, 418.1317; found 418.1319; HPLC purity: >95%. (UV 254 and 280 nm).

## Supplementary Material



## Notes

Ligand docking interactions (Figures S1–S8), docking score, and predicted pharmacokinetics and physiochemical properties (Tables S1 and S2), and the effects of Zinc13732787 and Chromanol 293B on KCNQ1 channel function (Figures S9 and S10), and details of Zinc13732787 (Figures S11–S27) (PDF)

## Abbreviations

KCNQ1: potassium voltage-gated channel subfamily Q member 1
KCNE1: potassium voltage-gated channel subfamily E regulatory subunit 1
I_Ks_: slow delayed rectifier potassium current
NSCs: neural stem cells
iPSC: induced pluripotent stem cells
TEVC: two-electrode voltage clamp
HTVS: high-throughput virtual screening
SP: standard precision
MM-GBSA: molecular mechanics generalized Born surface area
ADMET: absorption, distribution, metabolism, excretion, and toxicity
BBB: blood–brain barrier
hERG: Human ether-à-go-go-related gene
