# Supplementary material for: Discovery of Small-Molecule Inhibitors of the KCNQ1/Kv7.1 Potassium Channel with Virtual Screening and Functional Validation in Electrophysiological Assays and KCNQ1-Knockout Neural Stem Cells
Source: ACS Chem Neurosci. 2026 May 21;17(11):2203–18. doi: 10.1021/acschemneuro.6c00197 (PMC13237734; doi:10.1021/acschemneuro.6c00197)
Supplement: Supplementary file 1 [file cn6c00197_si_001.pdf]

Supplementary Information for the original article "Discovery of small-molecule inhibitors of the KCNQ1/Kv7.1 potassium channel with virtual screening and functional validation in electrophysiological assays and KCNQ1-knockout neural stem cells"

Kazi Asraful Alam , Josep Martí-Solans[#], Dorothea Schall[#], Sumit Kumar, Shahid Muhammad Iqbal, Knut Teigen, Simone Berkel, Daniela Mauceri, Bengt Erik Haug, Sara I Liin, Timothy Lynagh, Aurora Martinez[*] and Jan Haavik*

# Authors contributed equally

*Shared corresponding authors

Professor Jan Haavik, PhD, MD
Department of Biomedicine
University of Bergen, 5007 Bergen, Norway
Division of Psychiatry, Haukeland University Hospital, 5009 Bergen, Norway
E-mail: jan.haavik@uib.no

Professor Aurora Martinez, PhD
Department of Biomedicine
University of Bergen, 5007 Bergen, Norway
E-mail: aurora.martinez@uib.no

Author information

# Authors contributed equally.

Kazi Asraful Alam
Department of Biomedicine, University of Bergen, 5007 Bergen, Norway

Josep Martí-Solans
Michael Sars Centre, University of Bergen, 5006 Bergen, Norway

Dorothea Schall
Institute of Human Genetics, Heidelberg University, 69120 Heidelberg, Germany

Sumit Kumar
Department of Biomedicine, University of Bergen, 5007 Bergen, Norway
Department of Chemistry, University of Bergen, 5007 Bergen, Norway

Shahid Muhammad Iqbal
Michael Sars Centre, University of Bergen, 5006 Bergen, Norway

Knut Teigen
Department of Biomedicine, University of Bergen, 5007 Bergen, Norway

Simone Berkel
Institute of Human Genetics, Heidelberg University, 69120 Heidelberg, Germany

Daniela Mauceri
Department of Neurobiology, Interdisciplinary Centre for Neurosciences (IZN), Heidelberg University, 69120 Heidelberg, Germany
Institute of Anatomy and Cell Biology, Dept. Molecular and Cellular Neuroscience, University of Marburg, 35037 Marburg, Germany

Bengt Erik Haug
Department of Chemistry, University of Bergen, 5007 Bergen, Norway

Sara I Liin
Department of Biomedical and Clinical Sciences, Linköping University, 58185 Linköping, Sweden

Timothy Lynagh
Michael Sars Centre, University of Bergen, 5006 Bergen, Norway

Aurora Martinez*
Department of Biomedicine, University of Bergen, 5007 Bergen, Norway
Neuro-SysMed, Department of Neurology, Haukeland University Hospital, 5009 Bergen, Norway

Jan Haavik*
Department of Biomedicine, University of Bergen, 5007 Bergen, Norway
Division of Psychiatry, Haukeland University Hospital, 5009 Bergen, Norway

Table of Contents **Page no**

1	Computational section	S4-S7
2	Electrophysiological figures	S8-S9
3	Analytical data (NMR, HPLC and HRMS)	S10-S18
4	Tables: Docking scores and Predicted pharmacokinetics properties	**Error! Bookmark not defined.**9

Computational Section: S1-S8

In the Docking poses. The KCNQ1 structure, the subunits subunit A, D, G, and J are designated as I, II, III, and IV, respectively.

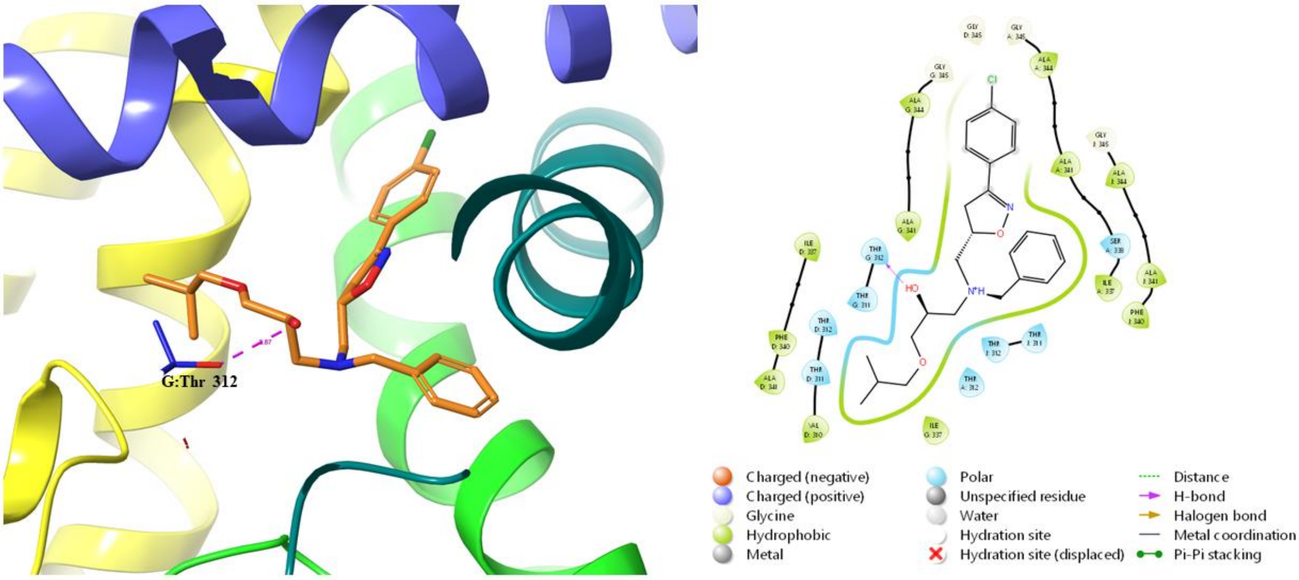

Figure S1: Zinc36145967 predicted pose with KCNQ1. Left panel show key residues having hydrogen bond interaction, and 2D display showing additional interaction at the Right panel.

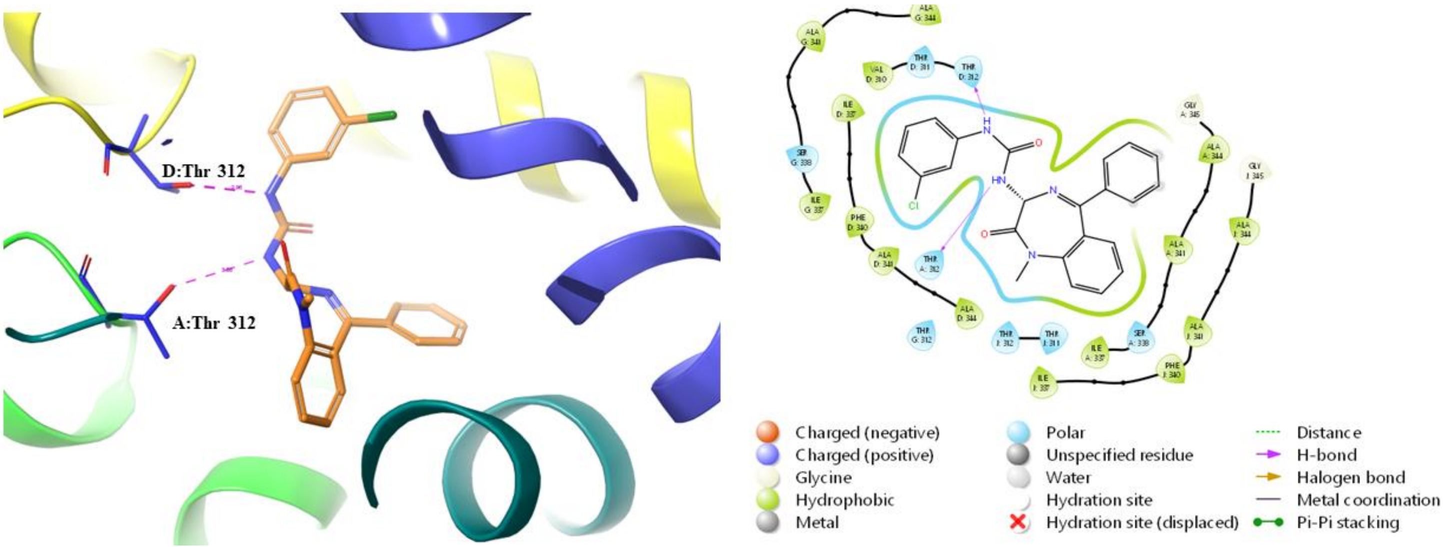

Figure S2: Zinc13732787 predicted pose with KCNQ1. Left panel show key residues having hydrogen bond interaction, and 2D display showing additional interaction at the Right panel.

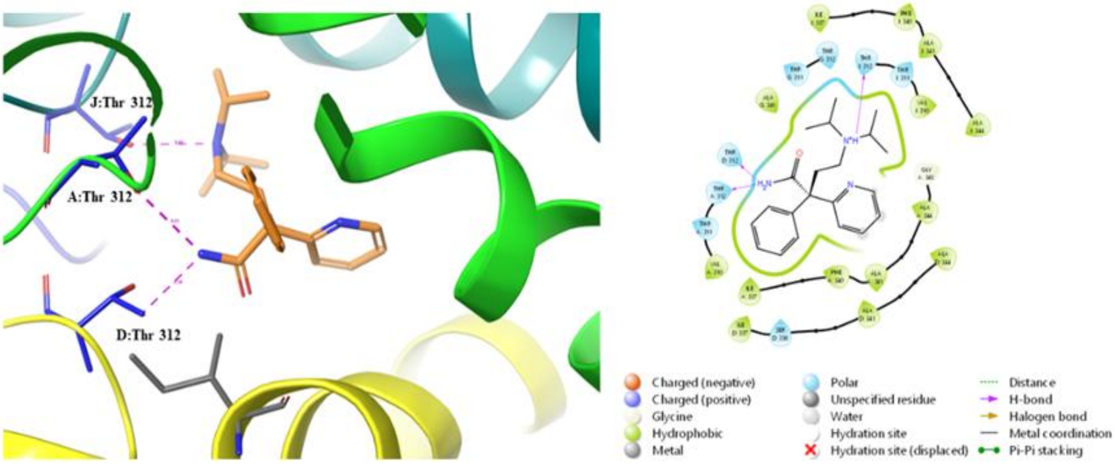

Figure S3: Predicted binding interaction of KCNQ1 with Disopyramide. Left panel show key residues having hydrogen bond interaction, and 2D display showing additional interaction at the Right panel.

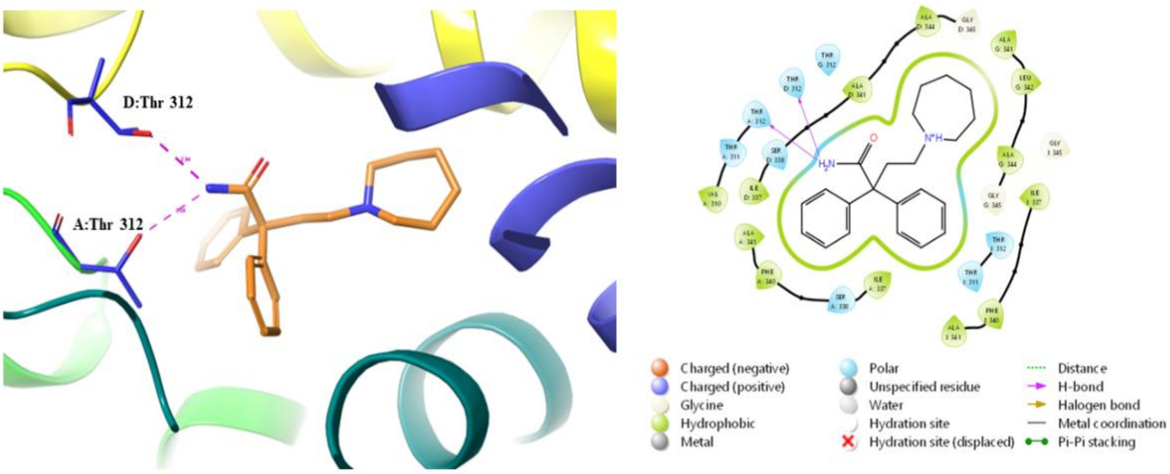

Figure S4: Zinc5849645 predicted pose with KCNQ1 . Left panel show key residues having hydrogen bond interaction, and 2D display showing additional interaction at the Right panel.

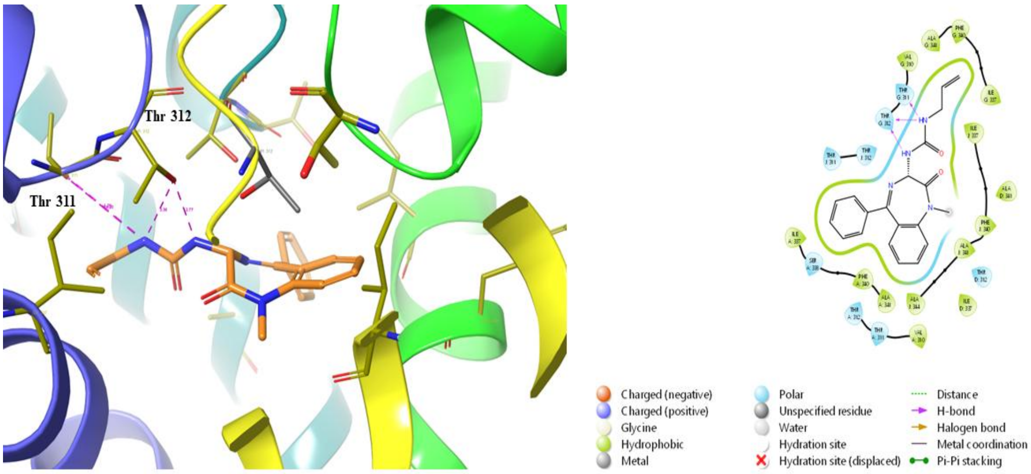

Figure S5: Zinc95929294 predicted pose with KCNQ1. The left side describes the docked conformation, while the right side of the figure illustrates 2D interaction

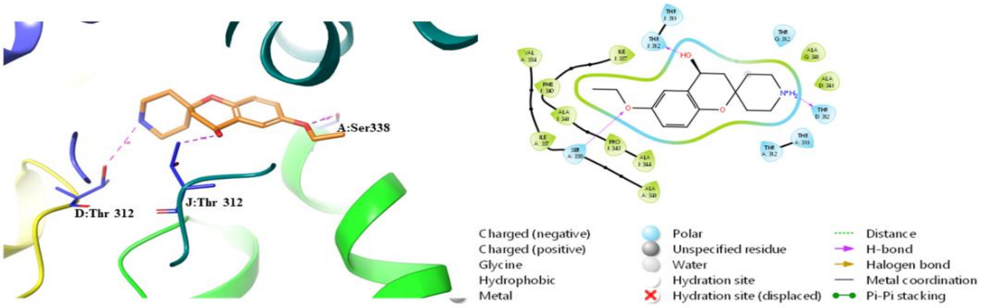

Figure S6: Zinc36141291predicted pose with KCNQ1. The left side describes the docked conformation, while the right side of the figure illustrates 2D interaction diagrams.

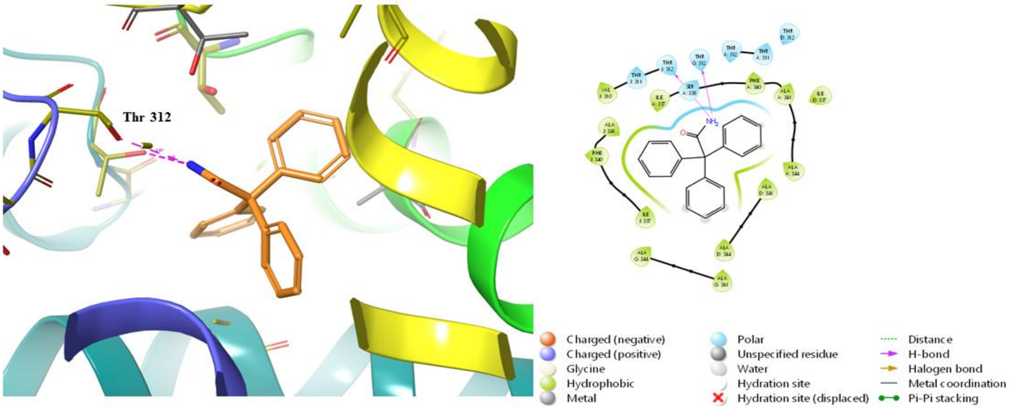

Figure S7: Zinc4017334 predicted pose with KCNQ1. The left side describes the docked conformation, while the right side of the figure illustrates 2D interaction diagrams.

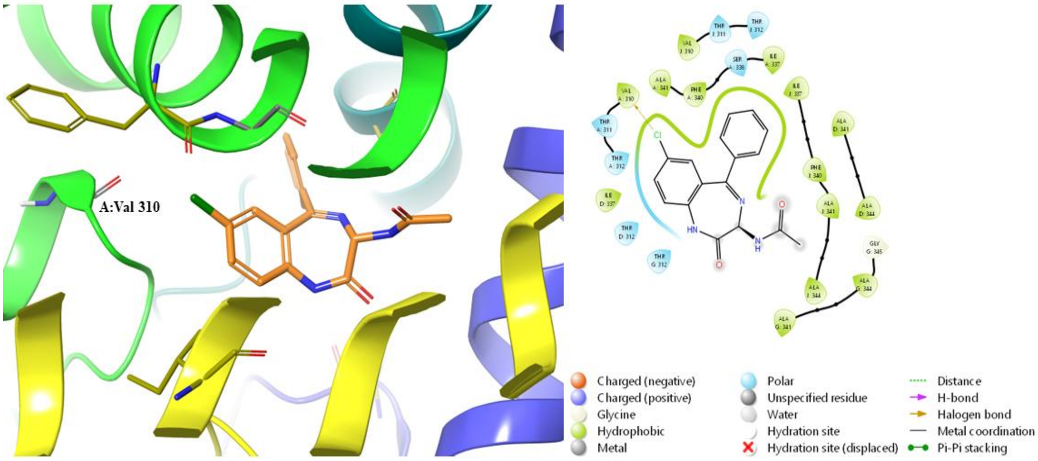

Figure S8: Zinc33819213 predicted pose with KCNQ1. The left side describes the docked conformation, while the right side of the figure illustrates 2D interaction diagrams.

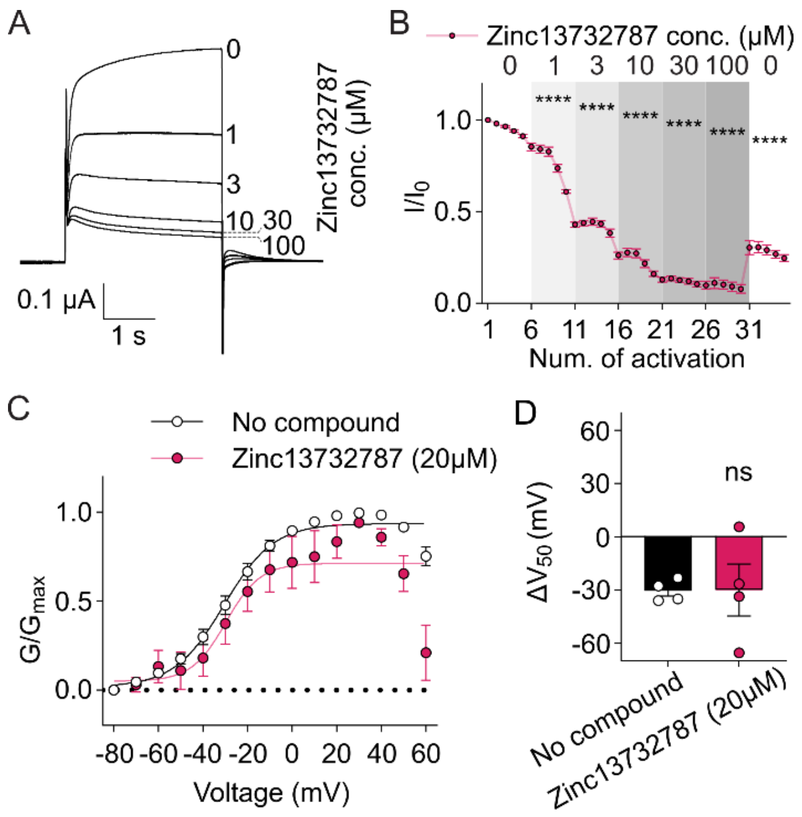

Figure S9: **Effect of Zinc13732787 on KCNQ1 Channel Function**. **A,** Representative K^+ current traces recorded from KCNQ1 channels elicited by voltage steps from -80 to +40 mV in oocytes exposed to the indicated concentrations (μM) of Zinc13732787. 6 ng of KCNQ1 of cRNA were injected into stage V/VI *Xenopus laevis* oocytes. **B,** Average normalized K^+ currents through KCNQ1 channels in response to voltage steps from -80 to +40 mV, recorded in the absence of Zinc13732787 and with increasing concentrations (10-second rest between activations). One-way ANOVA: **** $p < 0.0001$, n=3. **C,** Normalized tail current amplitude (G/G_{max}) plotted against membrane voltage (G–V curves) to illustrate the voltage dependence of activation. Depolarizing voltage pulses (2 s duration) ranged from -80 to +60 mV in 10 mV increments, followed by a step back to 0 mV for 1 s to elicit tail currents. **D,** V_{50} values derived

from the G–V curves. Data are presented as mean ± SEM. A paired t-test revealed no significant difference in V_{50} between the absence and presence of 20 µM Zinc13732787 ($P > 0.05$; $n = 4$).

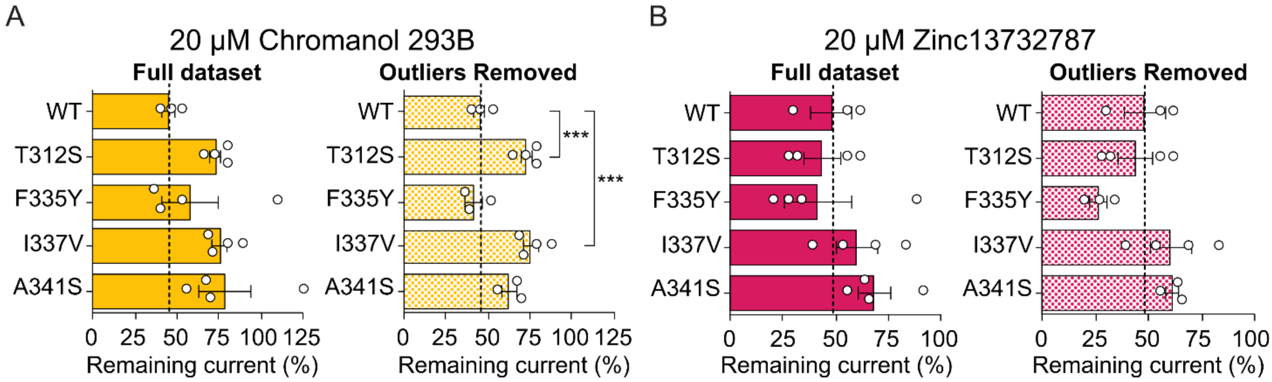

Figure S10: Using the full dataset, a one-way ANOVA showed no significant difference between the mutant channels treated with either compound and the WT ($F = 1.28$ for Chromanol 293B; $F = 1.02$ for Zinc13732787; $n = 1$-4). However, when we removed outliers and performed a one-way ANOVA followed by Tukey's HSD test, the Chromanol 293B-treated channels showed a significant difference ($F = 13.95$, ***$p < 0.005$), whereas the Zinc13732787-treated channels did not ($F = 5.22$).

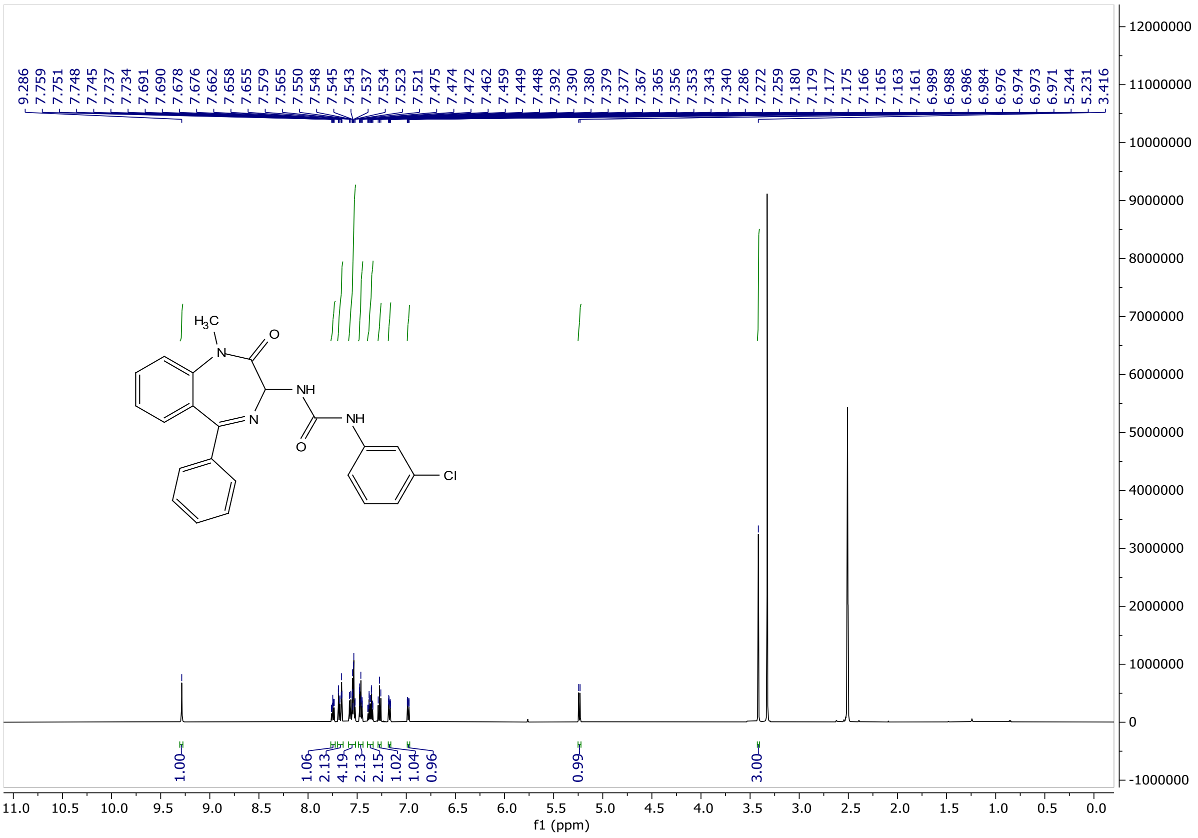

Figure S11. ^{1}H NMR spectrum of **2.**

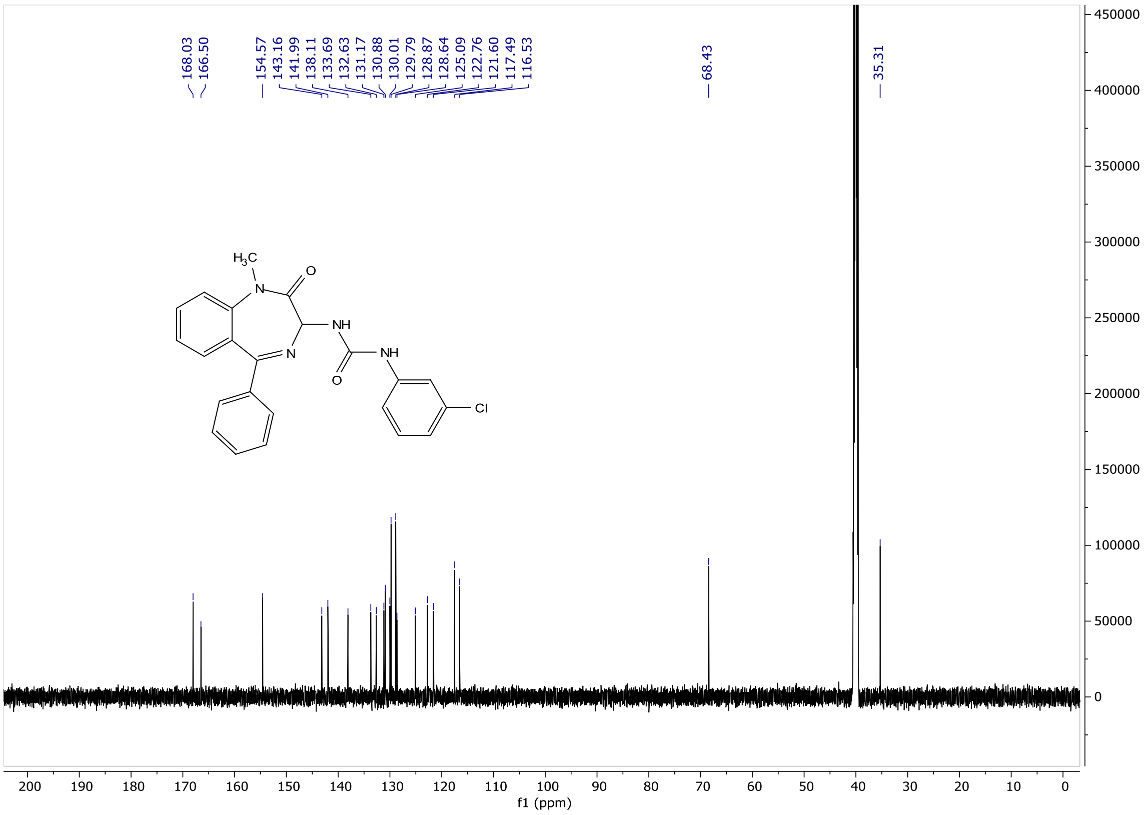

Figure S12. ^{13}C NMR spectrum of **2.**

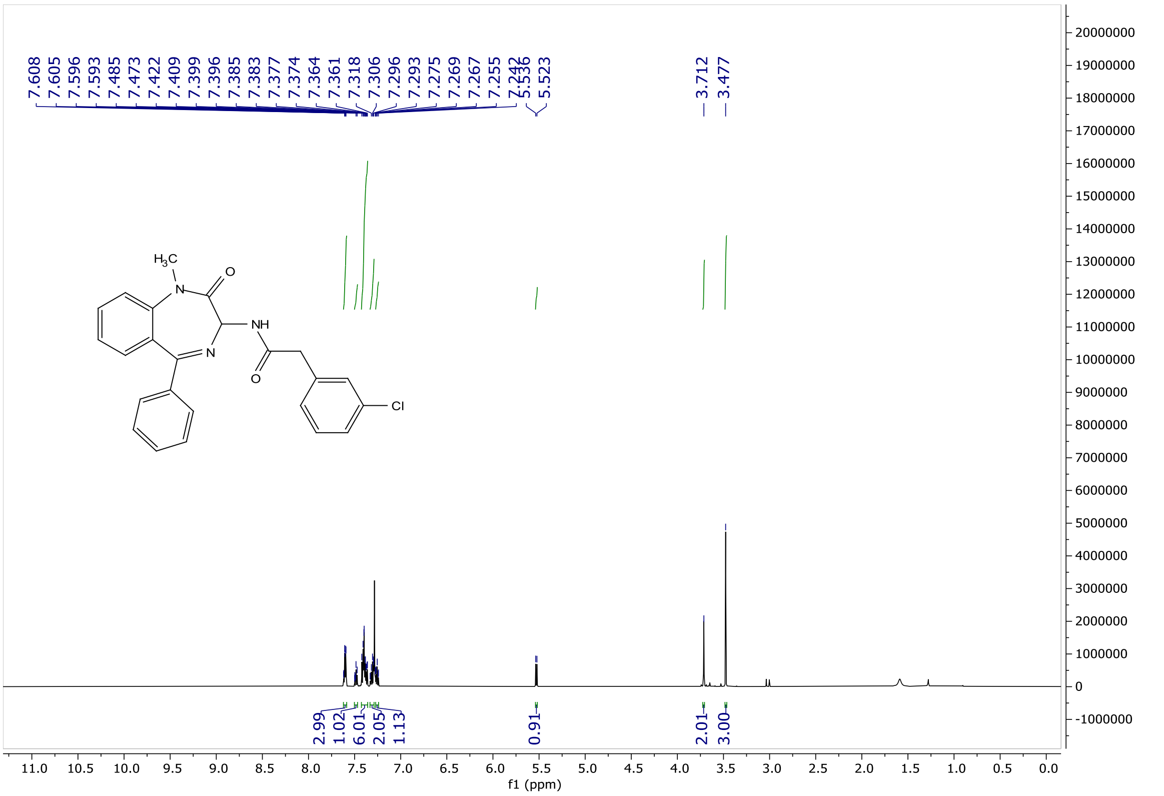

Figure S13. ^{1}H NMR spectrum of **3.**

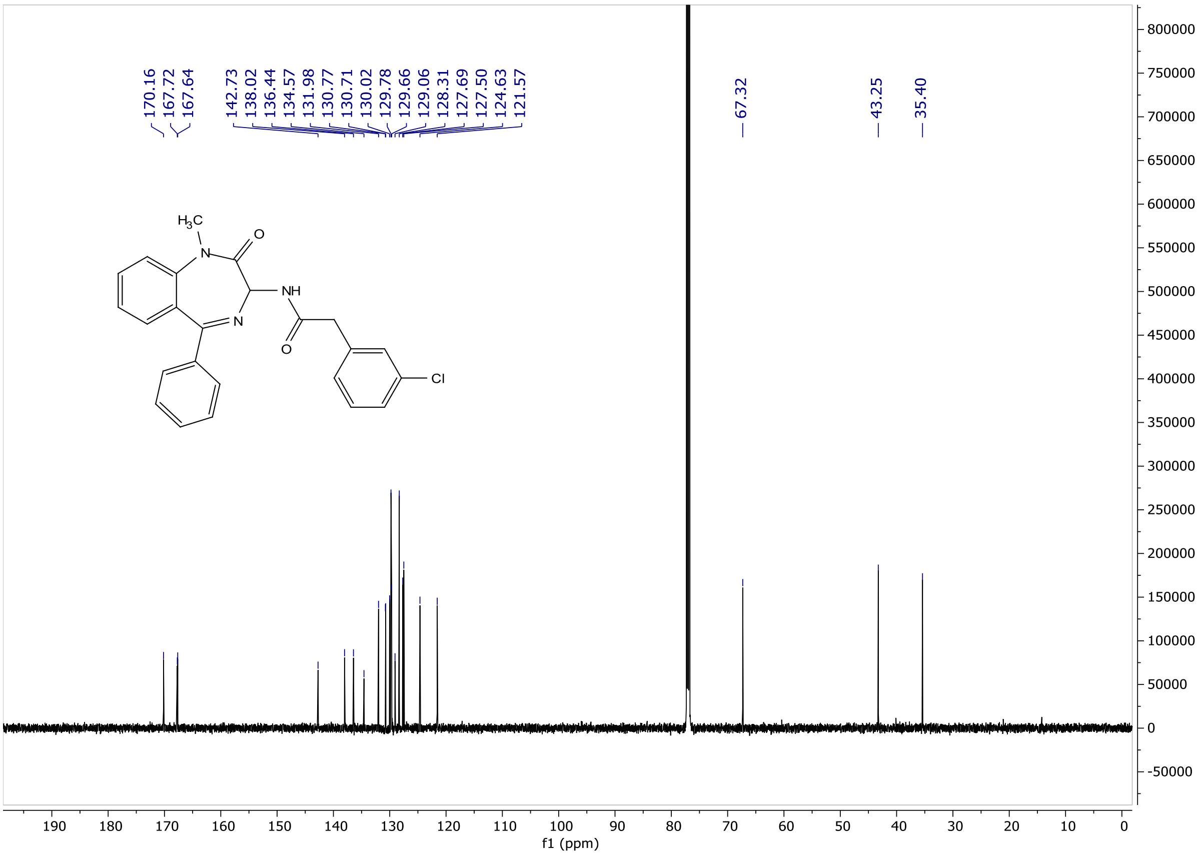

Figure S14. ^{13}C NMR spectrum of **3.**

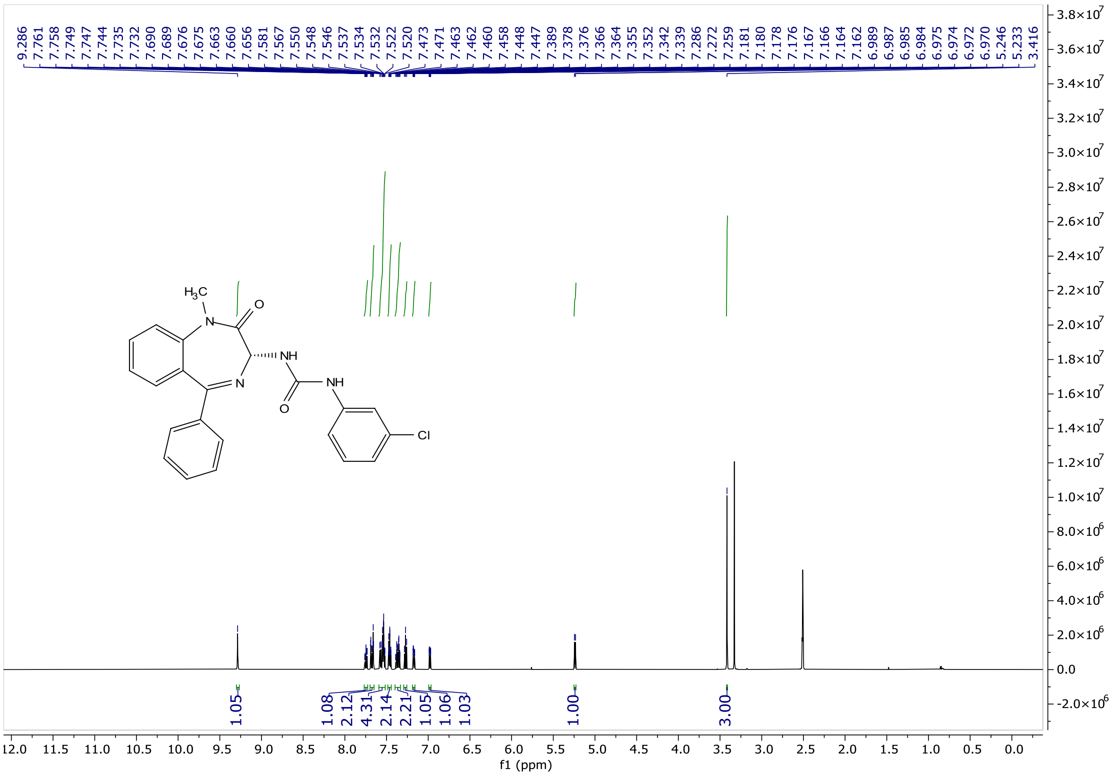

Figure S15. ^{1}H NMR spectrum of **(*R*)-2: Zinc 13732787.**

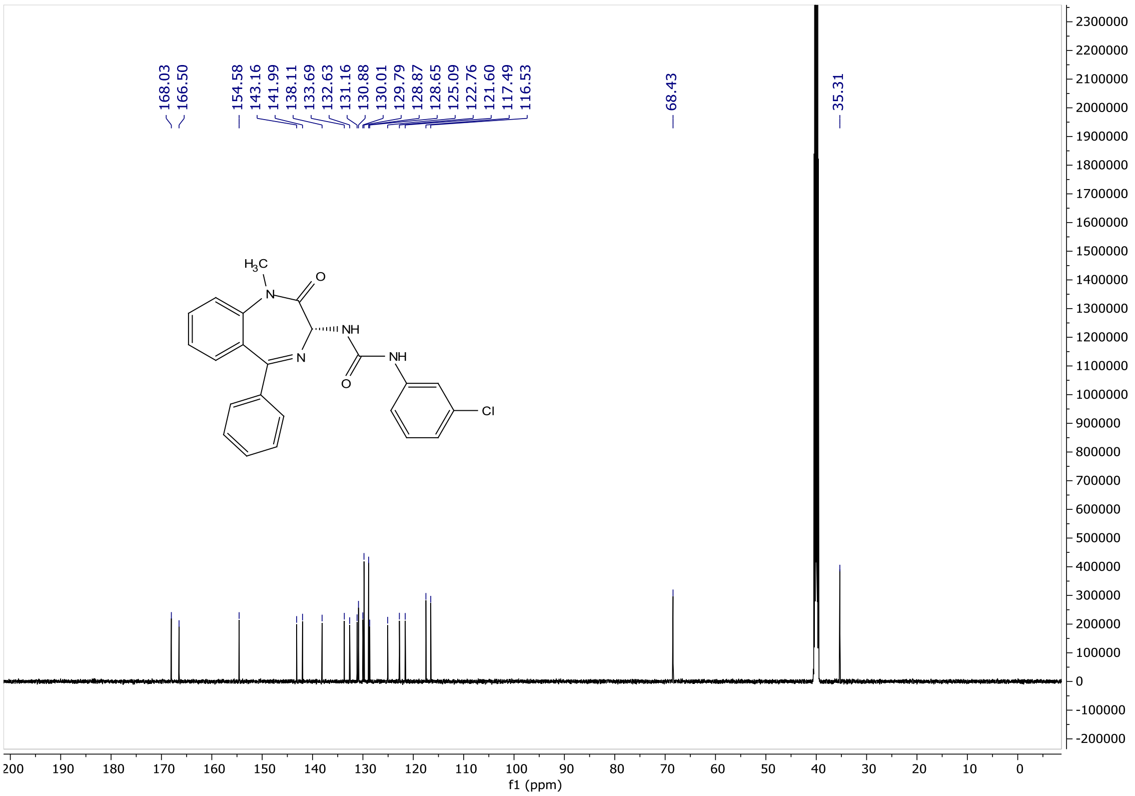

Figure S16. ^{13}C NMR spectrum of **(*R*)-2: Zinc 13732787**

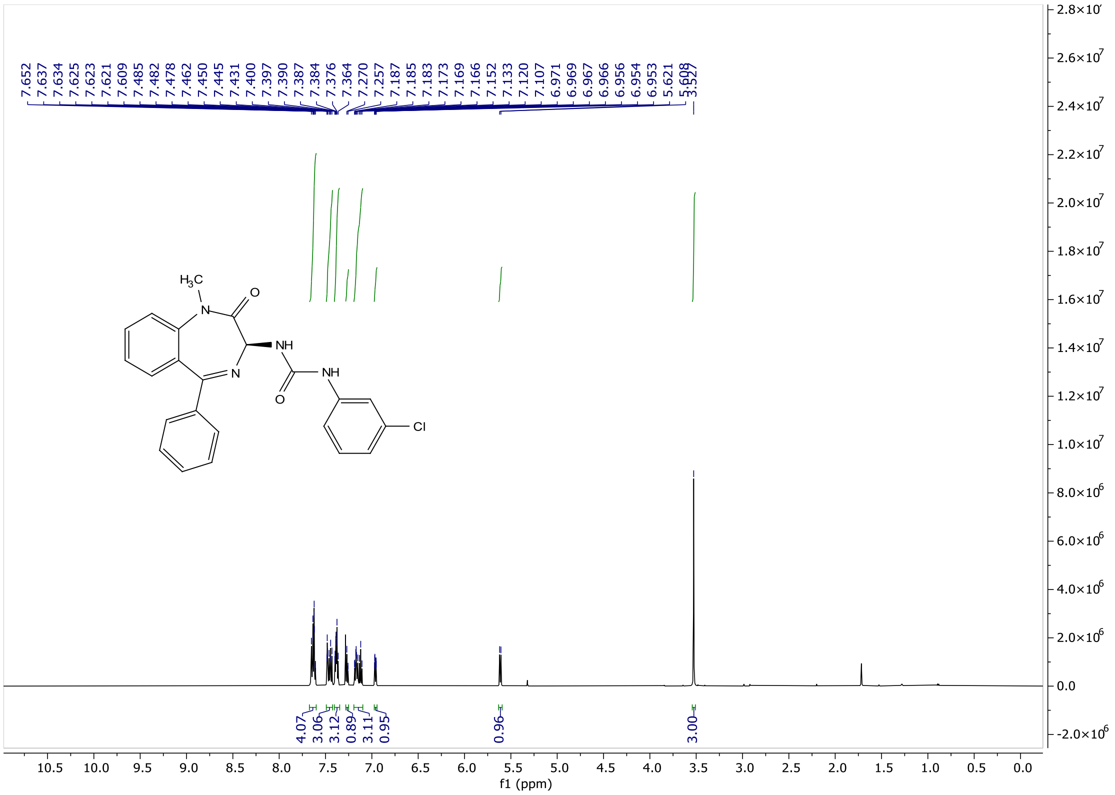

Figure S17. ^{1}H NMR spectrum of **(*S*)-2**.

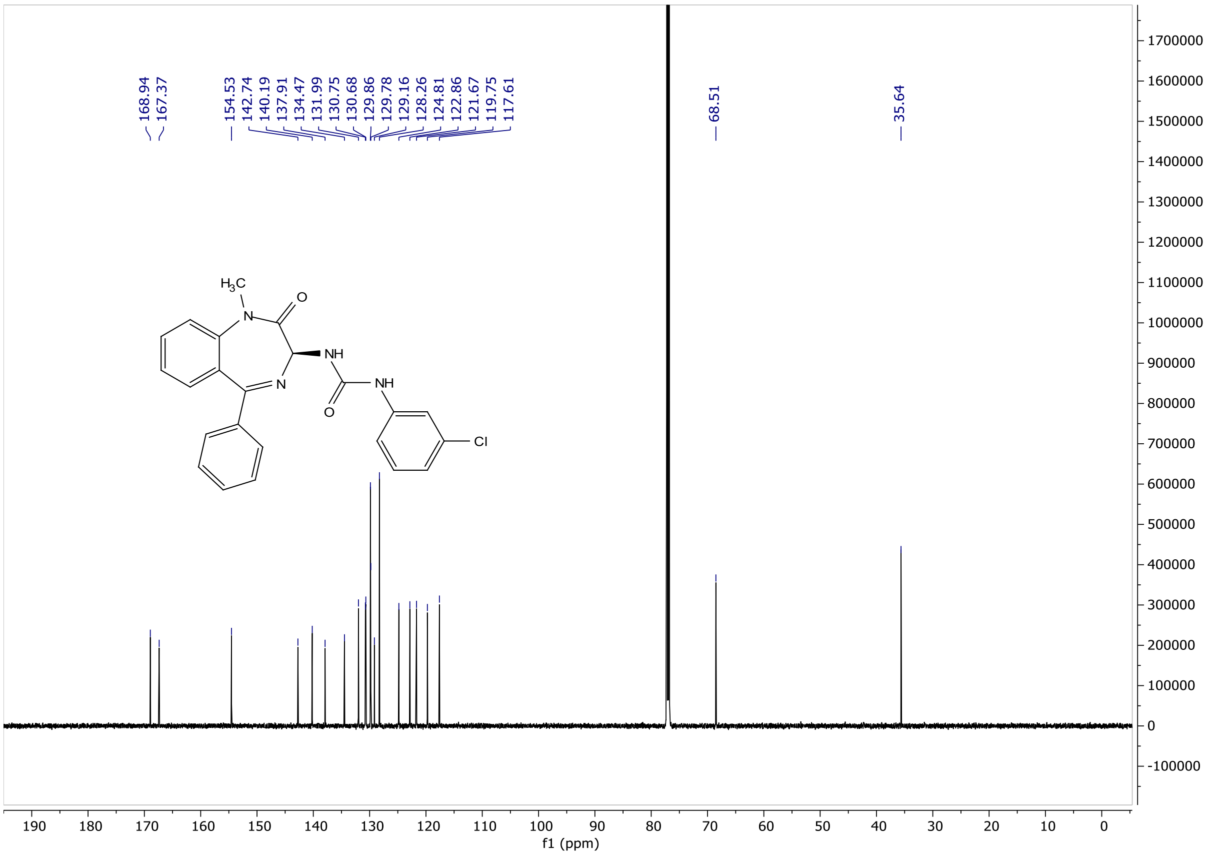

Figure S18. ^{13}C NMR spectrum of **(*S*)-2**.

HPLC Purity Analyses

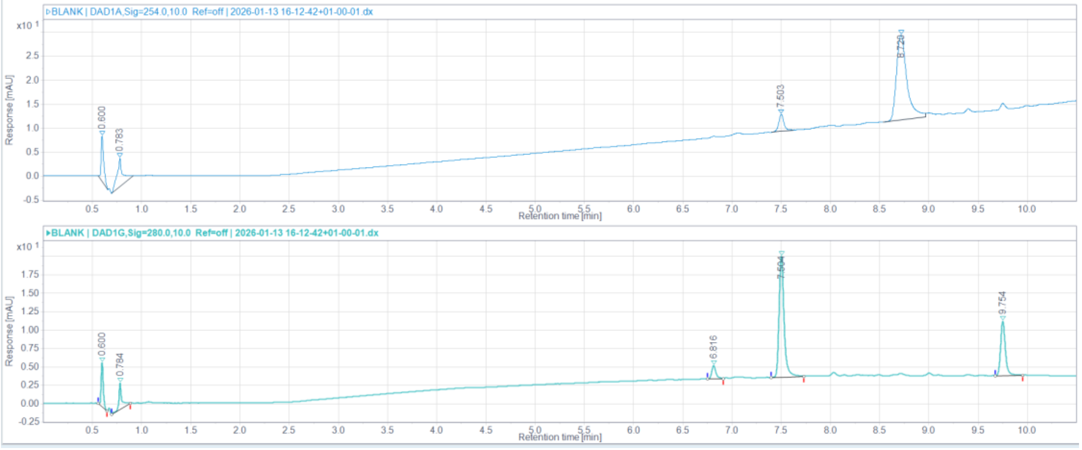

Figure S19. Blank run with detection at 254 nm (top panel) and at 280 nm (bottom panel). Impurities detected in the blank run have been omitted when calculating purity of each tested compound.

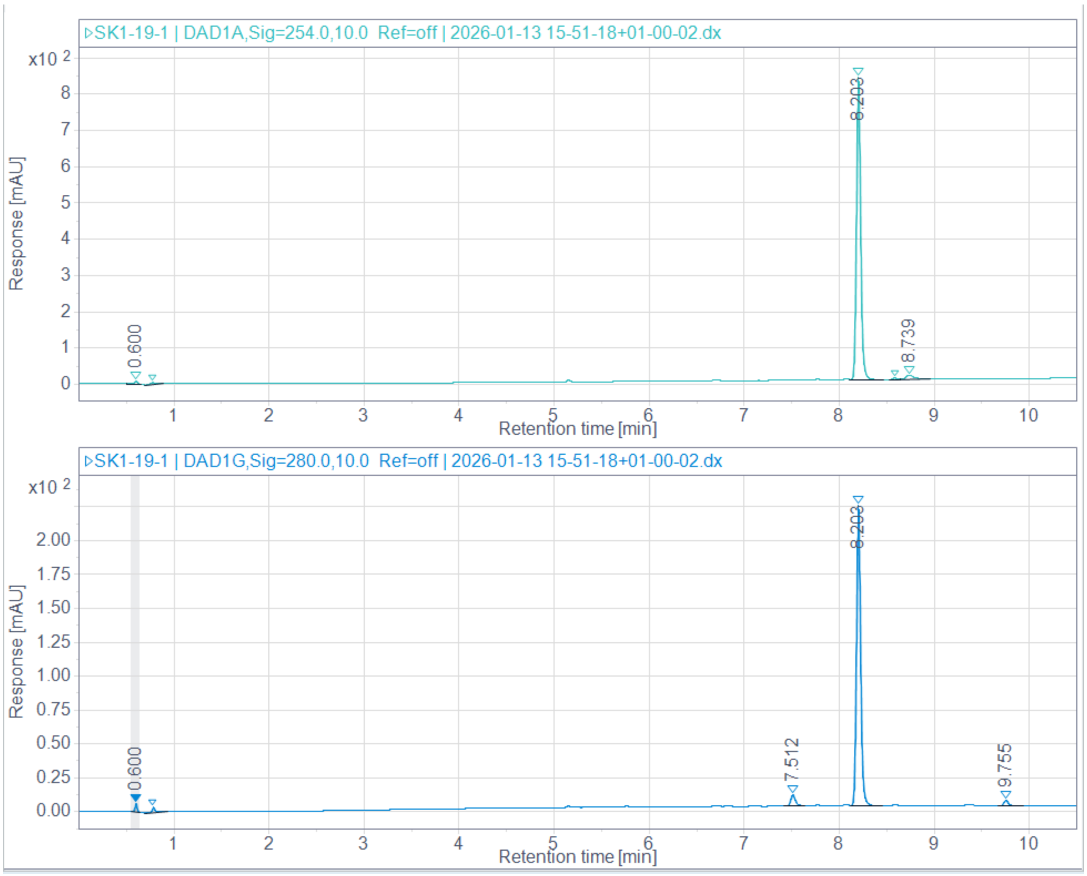

#	Name	Signal description	RT (min)	Area (mAU·s)	Area%	Height (mAU)	Height%	Amount	Concentr...	Start time (...	End tim...
1		DAD1A,Sig=254.0,10.0 Ref=off	0.600	28.355	1.078	10.613	1.23			0.510	0.661
2		DAD1A,Sig=254.0,10.0 Ref=off	0.782	22.410	0.852	5.899	0.68			0.700	0.900
3		DAD1A,Sig=254.0,10.0 Ref=off	8.203	2495.680	94.903	833.962	96.30			8.114	8.474
4		DAD1A,Sig=254.0,10.0 Ref=off	8.587	11.150	0.424	3.934	0.45			8.532	8.647
5		DAD1A,Sig=254.0,10.0 Ref=off	8.739	72.119	2.742	11.609	1.34			8.647	8.969

Figure S20. HPLC purity analysis of compound **2**.

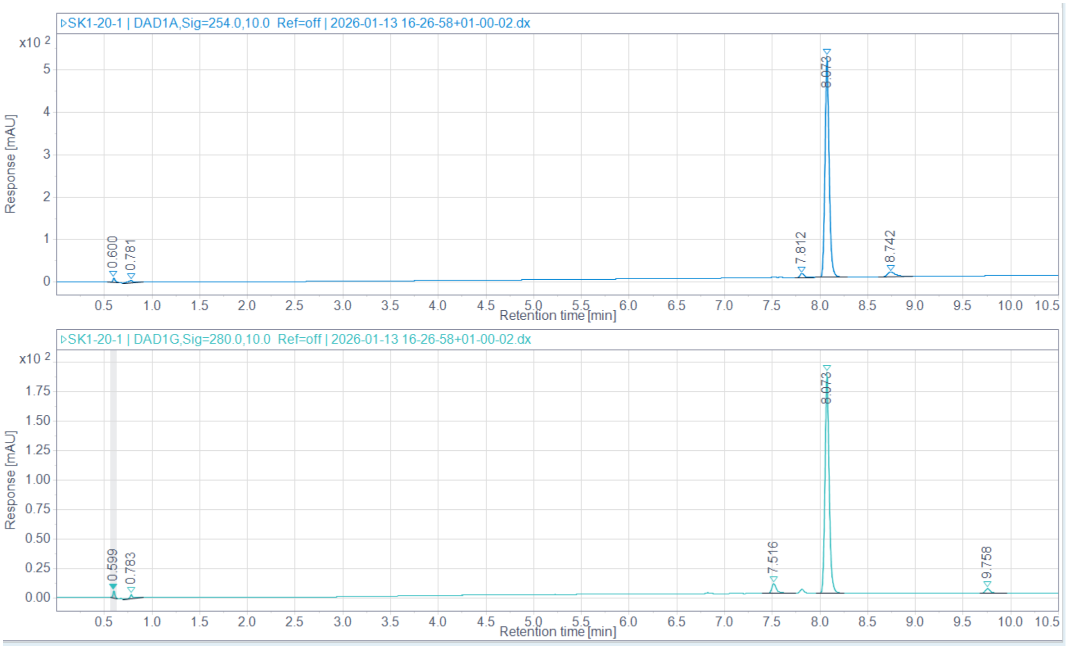

#	Name	Signal description	RT (min)	Area (mAU·s)	Area%	Height (mAU)	Height%	Amount	Concentr...	Start time (...	End tim...
1		DAD1A,Sig=254.0,10.0 Ref=off	0.600	20.433	1.192	9.906	1.78			0.535	0.659
2		DAD1A,Sig=254.0,10.0 Ref=off	0.781	23.868	1.392	5.976	1.07			0.700	0.915
3		DAD1A,Sig=254.0,10.0 Ref=off	7.812	28.458	1.660	9.341	1.68			7.742	7.940
4		DAD1A,Sig=254.0,10.0 Ref=off	8.073	1572.218	91.709	521.489	93.53			7.954	8.295
5		DAD1A,Sig=254.0,10.0 Ref=off	8.742	69.379	4.047	10.858	1.95			8.614	8.982

Figure S21. HPLC purity analysis of compound **3**.

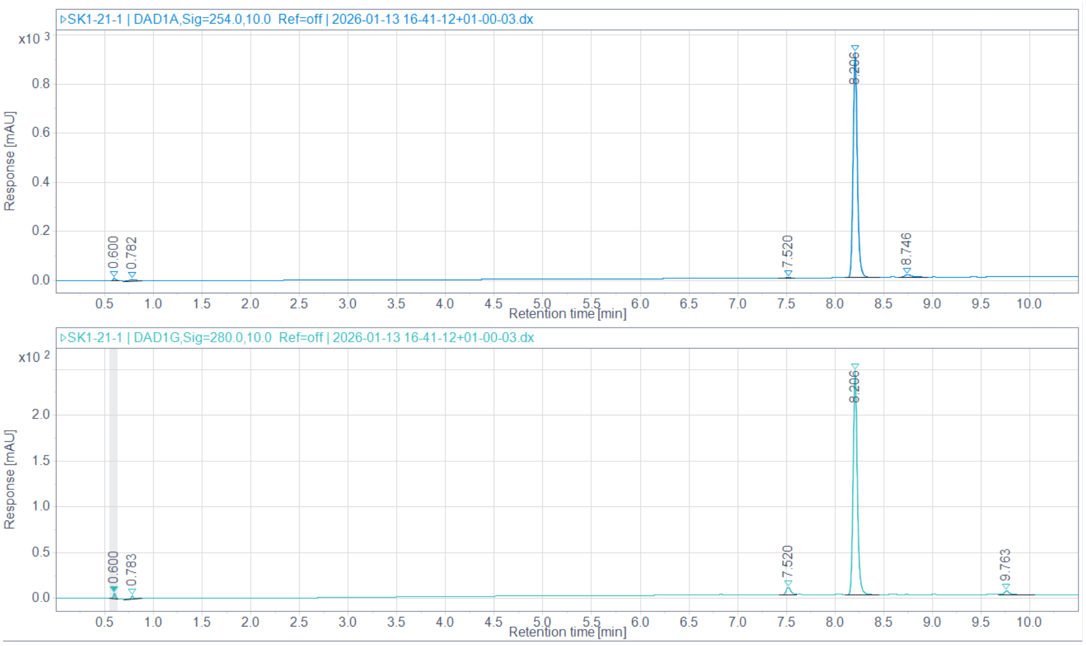

#	Name	Signal description	RT (min)	Area (mAU·s)	Area%	Height (mAU)	Height%	Amount	Concentr...	Start time (...	End tim...
1		DAD1A,Sig=254.0,10.0 Ref=off	0.600	17.223	0.597	9.226	0.98			0.571	0.658
2		DAD1A,Sig=254.0,10.0 Ref=off	0.782	21.000	0.728	5.735	0.61			0.700	0.889
3		DAD1A,Sig=254.0,10.0 Ref=off	7.520	6.278	0.218	1.780	0.19			7.421	7.591
4		DAD1A,Sig=254.0,10.0 Ref=off	8.206	2774.989	96.176	916.766	97.11			8.104	8.460
5		DAD1A,Sig=254.0,10.0 Ref=off	8.746	65.839	2.282	10.577	1.12			8.656	8.957

Figure S22. HPLC purity analysis of compound **(*R*)-2: Zinc 13732787.**

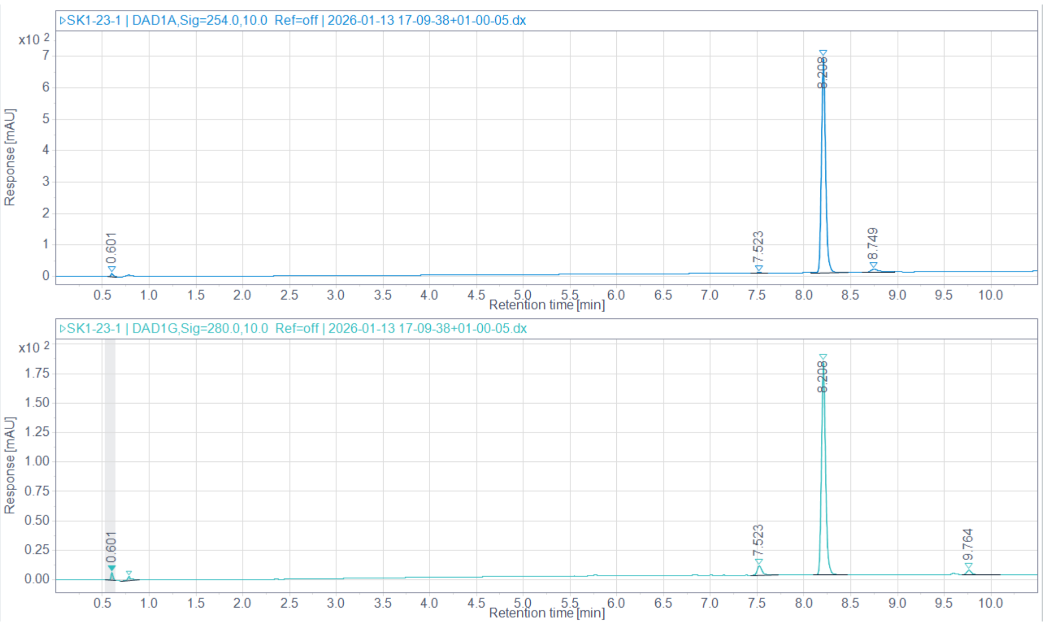

#	Name	Signal description	RT (min)	Area (mAU·s)	Area%	Height (mAU)	Height%	Amount	Concentr...	Start time (...	End tim...
1		DAD1A,Sig=254.0,10.0 Ref=off	0.601	18.241	0.838	9.491	1.34			0.562	0.657
2		DAD1A,Sig=254.0,10.0 Ref=off	7.523	6.560	0.301	1.771	0.25			7.434	7.620
3		DAD1A,Sig=254.0,10.0 Ref=off	8.208	2080.781	95.540	687.046	96.94			8.081	8.483
4		DAD1A,Sig=254.0,10.0 Ref=off	8.749	72.334	3.321	10.452	1.47			8.629	8.977

Figure S23. HPLC purity analysis of compound **(*S*)-2**

LC-HRMS Analyses:

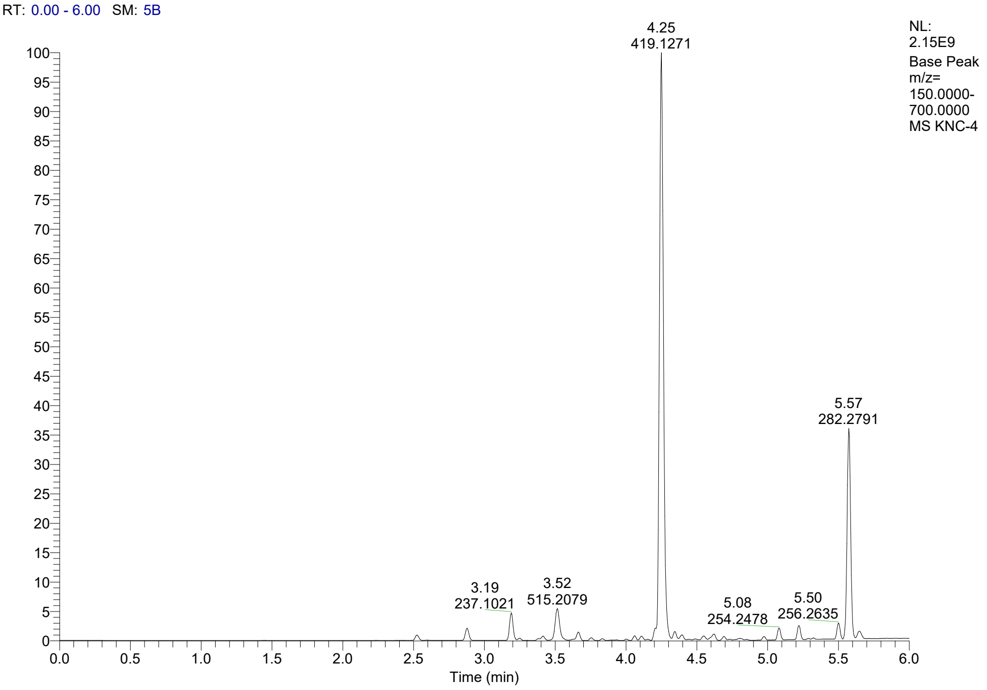

Figure S24. LC-HRMS analysis of compound **2**. Peak at $R_t = 4.25$ corresponds to $[M+H]^+$ for $C_{23}H_{20}{}^{35}ClN_4O_2{}^+$ (calcd $m/z = 419.1270$).

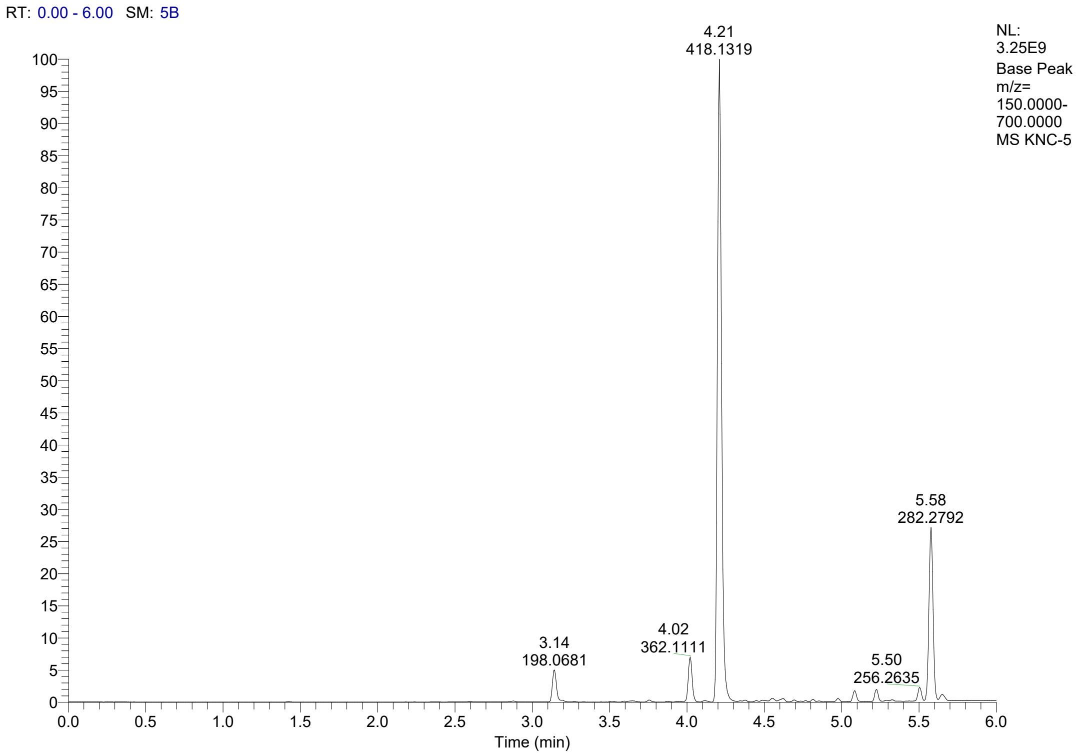

Figure S25. LC-HRMS analysis of compound **3**. Peak at R_t = 4.21 corresponds to $[M+H]^+$ for $C_{24}H_{21}{}^{35}ClN_3O_2{}^+$ (calcd *m/z* = 418.1317).

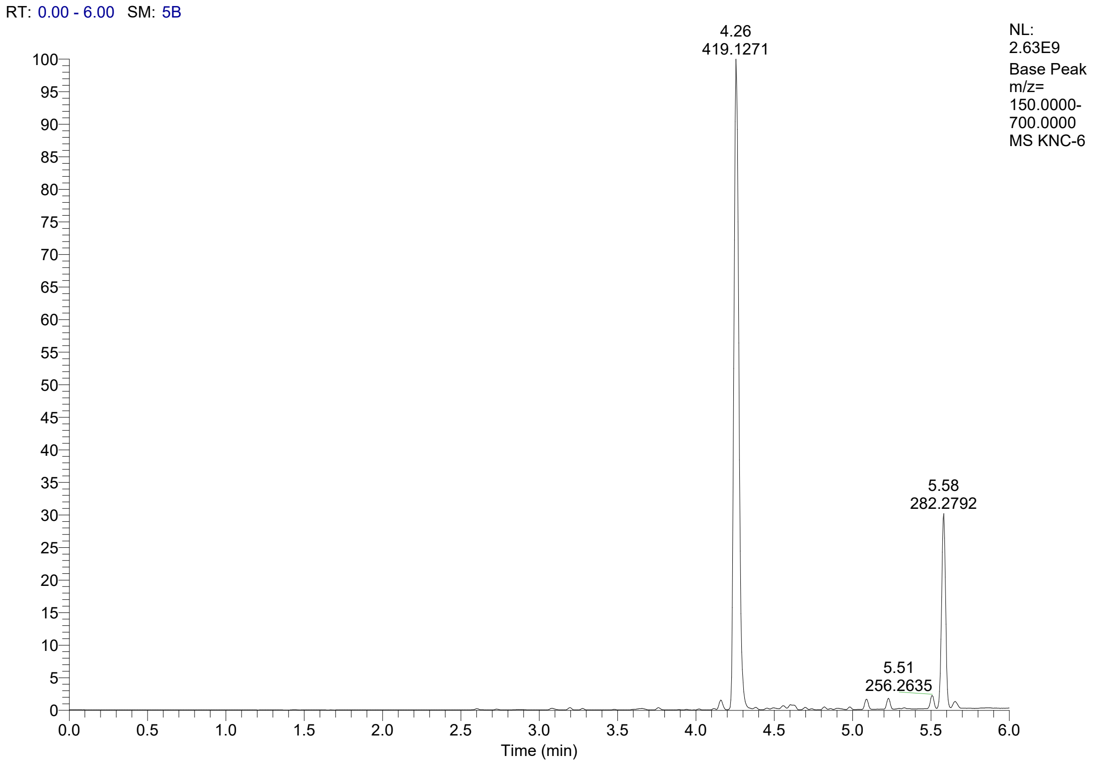

Figure S26. LC-HRMS analysis of compound **(*R*)-2: Zinc13732787**. Peak at R_t = 4.26 corresponds to $[M+H]^+$ for $C_{23}H_{20}{}^{35}ClN_4O_2{}^+$ (calcd *m/z* = 419.1270).

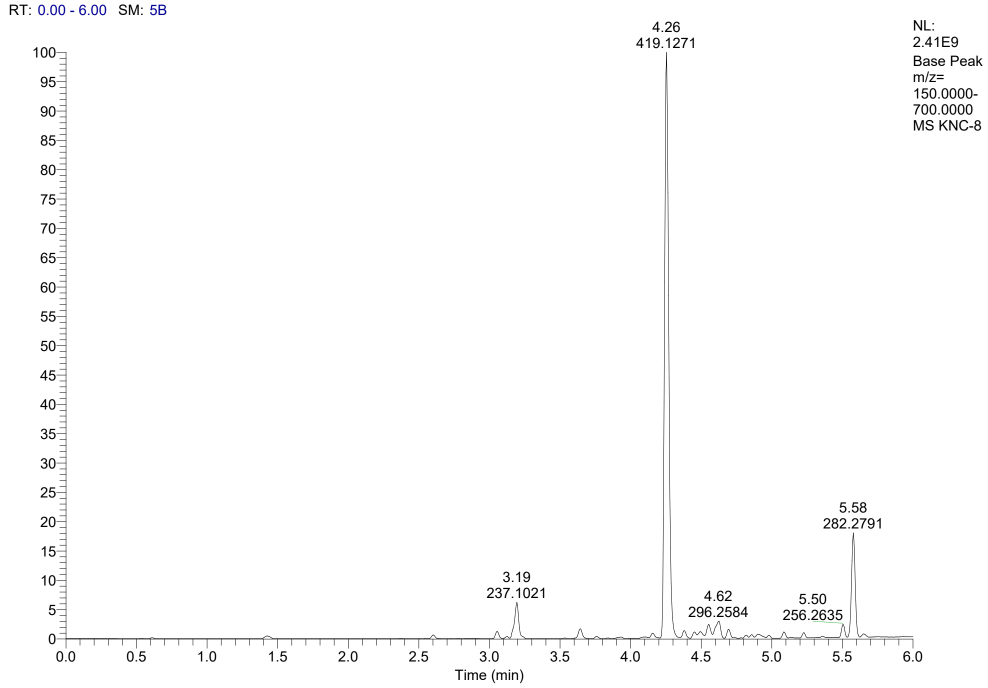

Figure S27. LC-HRMS analysis of compound **(*S*)-2**. Peak at R_t = 4.26 corresponds to $[M+H]^+$ for $C_{23}H_{20}{}^{35}ClN_4O_2{}^+$ (calcd *m/z* = 419.1270).

Table S1. HTVS screening of ligands and standard precision (SP) docking score with relative free energy of KCNQ1-apo structure.

Sl. No.	Ligands Id	Docking score (kcal/mol)	Prime MMGBSA-dG-binding energy	Mol. Weight (g/mol)
1	Zinc36145967	-8.61	-36.88	430
2	Zinc13732787	-8.46	-26.18	418
3	Disopyramide	-8.02	-23.74	339
4	Zinc5849645	-7.96	-33.22	336
5	Zinc95929294	-7.17	-29.13	348
6	Zinc36141291	-6.93	-29.95	263
7	Zinc4017334	-6.02	-29.77	287
8	Zinc33819213	-5.93	-39.35	327

Table S2. Predicted pharmacokinetics and physicochemical properties of selected KCNQ1 ligands, including gastrointestinal (GI) absorption, blood-brain barrier (BBB) permeability, P-glycoprotein (P-gp), CYP1A2 interactions, hERG inhibition, hydrogen bonding capacity, and topological polar surface area (TPSA).

Sl. No.	**Ligand Id**	**GI absorption**	**BBB**	**P-gp**	**CYP1A2**	**hERG inhibition**	**H-bond acceptors**	**H-bond donors**	**TPSA(Å^2)**
1	Zinc36145967	High	Yes	No	No	safe	5	1	54.29
2	Zinc13732787	High	Yes	No	No	safe	3	2	73.20
3	Disopyramide	High	Yes	No	No	safe	3	1	59.22
4	Zinc5849645	High	Yes	Yes	No	safe	2	1	46.33
5	Zinc95929294	High	No	No	No	safe	3	2	73.80
6	Zinc36141291	High	Yes	Yes	No	safe	4	2	50.72
7	Zinc4017334	High	Yes	No	Yes	safe	1	1	43.09
8	Zinc33819213	High	Yes	No	No	safe	3	2	70.56
9	Compound 3	High	Yes	No	No	safe	3	1	61.77
